# Incretin-based injectable strategies versus intensified insulin for treatment intensification and simplification in type 2 diabetes: a systematic review and meta-analysis

**DOI:** 10.1007/s40200-026-02089-x

**Published:** 2026-09-26

**Authors:** Talha Mahmood, Ioanna Myrtziou, Ioannis Kanakis

**Affiliations:** 1 Chester Medical School, Faculty of Health, Medicine and Society, University of Chester, Chester, CH1 4BJ UK; 2 School of Pharmacy and Biomolecular Sciences, Faculty of Health, Innovation, Technology and Science, Liverpool, L3 3AF UK; 3 Department of Musculoskeletal & Ageing Science, Institute of Life Course & Medical Sciences, University of Liverpool, Liverpool, L7 8TX UK

**Keywords:** Type 2 diabetes, GLP-1 receptor agonist, Tirzepatide, Incretin, Hypoglycaemia, Insulin intensification, Meta-analysis

## Abstract

**Purpose:**

Prandial insulin intensification in Type 2 Diabetes (T2D) improves glycaemia but increases regimen complexity, weight gain, and hypoglycaemia risk. Our aim was to evaluate if Incretin-based injectable strategies offer a lower-burden alternative across intensification and simplification pathways.

**Methods:**

PubMed/MEDLINE, CENTRAL, Scopus, and ClinicalTrials.gov were searched to November 2025 for Randomised Controlled Trials (RCT) comparing incretin-based injectable regimens with intensified insulin strategies in adults with T2D. Primary outcomes were HbA1c change and trial-defined hypoglycaemia. Secondary outcomes included body weight, HbA1c target achievement, severe hypoglycaemia, insulin dose, gastrointestinal adverse events, and adverse-event withdrawals. Random or fixed-effects models were applied where appropriate, with subgroup analyses by regimen and by intensification versus simplification design.

**Results:**

Eighteen RCTs were included. Incretin-based regimens significantly reduced HbA1c as compared to intensified insulin (MD -0.19%, 95% CI: -0.34 to -0.05, *P* = 0.01) and also increased HbA1c target achievement (RR 1.27, 95% CI: 1.05 to 1.54, *P* = 0.01). Effects differed by regimen and design: tirzepatide produced the largest HbA1c reduction, fixed-ratio or once-weekly combination regimens showed similar HbA1c efficacy, and simplification trials generally preserved glycaemic control. Incretin-based regimens decreased trial-defined hypoglycaemia (RR 0.48, 95% CI: 0.35 to 0.66, *P* = 0.00001), severe hypoglycaemia (RR 0.32, 95% CI: 0.19 to 0.51, *P* = 0.00001), and body weight (MD -4.65 kg, 95% CI: -5.85 to -3.44, *P* = 0.00001). Gastrointestinal adverse events were more frequent with incretin-based regimens.

**Conclusion:**

Incretin-based injectable strategies are a favourable alternative to intensified insulin when hypoglycaemia, weight gain, and treatment burden are priorities, but benefits differ between intensification and simplification settings.

**Supplementary Information:**

The online version contains supplementary material available at https://doi.org/10.1007/s40200-026-02089-x.

## Introduction

Diabetes mellitus remains a major global non-communicable disease, with an estimated 589 million adults aged 20–79 years living with diabetes in 2024 [1]. The International Diabetes Federation further estimated approximately 3.4 million diabetes-attributable deaths among adults aged 20–79 years and USD 1.015 trillion in global diabetes-related health expenditure in 2024 [2]. Type 2 diabetes (T2D) is progressive and often requires sequential treatment intensification as glycaemic control deteriorates [3]. The UK Prospective Diabetes Study (UKPDS) evidence demonstrated both the microvascular benefits of intensive glycaemic control and a graded relationship between HbA1c and diabetes-related complications [4, 5]. Therefore, contemporary guidance emphasises on individualised HbA1c targets that balance glycaemic benefit against hypoglycaemia risk, comorbidity, life expectancy, and treatment burden [3, 6].

When injectable therapy is required, basal insulin is commonly used to address fasting hyperglycaemia; however, persistent HbA1c elevation despite basal insulin titration may require treatment directed at postprandial glucose [3, 7]. Conventional intensification strategies include basal-plus, basal-bolus, and premixed or biphasic insulin regimens. Although effective in reducing HbA1c, these approaches increase injection frequency, glucose-monitoring requirements, dose-adjustment complexity, hypoglycaemia risk, and insulin-associated weight gain [3, 7]. Insulin therapy is frequently perceived as restrictive, and concerns about hypoglycaemia may limit treatment adherence and intensification [8]. Delayed intensification is well documented: in a UK cohort of 81,573 adults treated with oral glucose-lowering drugs, a large proportion of patients remained above the HbA1c thresholds for several years before treatment escalation, including insulin initiation [9].

Incretin-based injectable strategies provide a mechanistically and clinically plausible alternative to prandial insulin escalation. GLP-1 receptor agonists (GLP-1RAs) lower glucose by stimulating glucose-dependent insulin secretion, suppressing glucagon secretion, and delaying gastric emptying; they also reduce appetite, energy intake, and body weight [10, 11]. Current consensus guidance recognises adding a GLP-1RA to basal insulin as an alternative to mealtime insulin intensification in adults who remain above individualised glycaemic targets despite basal insulin titration [3, 7]. The evidence base encompasses GLP-1RAs administered separately alongside basal insulin, fixed-ratio basal insulin/GLP-1RA combinations such as insulin degludec/liraglutide and insulin glargine/lixisenatide, dual incretin agonist regimens such as tirzepatide plus basal insulin, and the once-weekly fixed-ratio combination of insulin icodec and semaglutide (IcoSema) [12–15].

Direct comparative RCTs span two distinct clinical pathways. DUAL VII, SURPASS-6, and COMBINE 3 studies evaluated intensification after basal insulin by comparing an incretin-containing strategy with a prandial-insulin-based regimen [13–15]. By contrast, the relevant comparison in the BEYOND trial evaluated simplification from established basal-bolus insulin to a once-daily basal insulin/GLP-1RA fixed-ratio combination versus continued basal-bolus therapy [16]. Although the formulations differed, all evaluated whether an incretin-containing injectable strategy could avoid or replace prandial-insulin intensification.

Basal insulin/GLP-1RA strategies can achieve glycaemic efficacy comparable to basal-plus or basal-bolus insulin while generally producing less hypoglycaemia and more favourable body-weight outcomes; fixed-ratio combinations can also reduce injection burden [3, 12, 14]. Previous meta-analyses assessed GLP-1RA/insulin combinations versus basal-plus or basal-bolus insulin but predated BEYOND, SURPASS-6, COMBINE 3, and the resulting expansion of simplification, dual-incretin, and once-weekly combination evidence [12, 13, 15, 16]. Distinguishing intensification from simplification is important because the former seeks additional glycaemic lowering after basal insulin, whereas the latter aims to reduce treatment burden while maintaining glycaemic control in people already receiving basal-bolus or multiple daily injection regimens.

Accordingly, this systematic review and meta-analysis aimed to evaluate whether, in adults with T2D requiring injectable intensification or simplification, incretin-based injectable strategies, compared with intensified insulin strategies, improve glycaemic control and patient-relevant safety and tolerability outcomes, particularly HbA1c, hypoglycaemia, body weight, insulin dose, gastrointestinal adverse events, and withdrawals due to adverse events.

## Methods

This systematic review and meta-analysis followed a prespecified protocol defining eligibility criteria, outcomes, and synthesis methods and was reported in accordance with the Preferred Reporting Items for Systematic Reviews and Meta-Analyses (PRISMA) 2020 statement [17]. The protocol was not prospectively registered.

### Search strategy

PubMed/MEDLINE, the Cochrane Central Register of Controlled Trials (CENTRAL) via the Cochrane Library, Scopus, and ClinicalTrials.gov were searched from inception to 25 November 2025. Reference lists of included trials and relevant systematic reviews were screened manually. Strategies combined controlled vocabulary, where available, and free-text terms for T2D, incretin-based injectable therapies, intensified insulin regimens, and randomised trials. No publication-date restrictions were applied. English-language and human-study limits were used in PubMed/MEDLINE and Scopus; no additional interface limits were applied to CENTRAL. Complete strategies, dates, filters, and record counts are provided in Supplementary Table S1.

### Study selection and eligibility criteria

Eligibility was defined using the Population, Intervention, Comparator, Outcomes, and Study design (PICOS) framework. Parallel-group RCTs were eligible if they enrolled adults with type 2 diabetes undergoing injectable treatment intensification or simplification, directly compared an incretin-based injectable strategy with an intensified-insulin strategy, and had at least 12 weeks of follow-up. Intensification studies evaluated treatment escalation, generally after inadequate control with basal insulin. Simplification studies evaluated withdrawal or replacement of prandial insulin in participants receiving basal-bolus insulin or multiple daily injections.

Eligible interventions were: a GLP-1 receptor agonist (GLP-1RA) administered separately alongside basal insulin; a fixed-ratio basal insulin/GLP-1RA combination; or a dual incretin agonist or related insulin-incretin co-formulation when adult T2D data were separately available. Eligible comparators were basal-plus insulin (basal insulin plus one prandial injection), basal-bolus insulin (basal insulin plus two or more prandial injections), and premixed or biphasic insulin used as an intensified strategy. Non-randomised and crossover studies, paediatric studies, studies of Type 1 or gestational diabetes, studies with less than 12 weeks of follow-up, and trials without an eligible intervention-comparator comparison were excluded. Studies were not excluded solely because data for a particular outcome were unavailable in a poolable format; eligible studies were retained in the systematic review, and relevant non-poolable findings were synthesised narratively.

Records were imported into EndNote 21 and deduplicated [18]. Two reviewers (TM and IM) independently screened titles and abstracts and assessed potentially eligible full texts. Disagreements were resolved by consensus, with third-reviewer (IK) adjudication when required. Reasons for full-text exclusion were recorded, and multiple reports of the same trial were collated under one study identifier.

### Data extraction and outcomes

Two reviewers (TM and IM) independently extracted data using a standardised form, with disagreements resolved by consensus. Extracted information included study and participant characteristics, eligibility criteria, intervention and comparator regimens, follow-up, outcome definitions, analysis population, funding and conflicts of interest, and numerical outcome data. Values were cross-checked against primary publications, supplementary materials, secondary reports, and trial-register records where available. Primary publications were prioritised for efficacy outcomes; supplementary and registry records were used to verify methods, safety outcomes, prespecified outcomes, and incomplete data. Study-to-report and study-to-analysis mappings are provided in Supplementary Table S2.

The primary efficacy outcome was change in glycated haemoglobin (HbA1c, percentage points) from baseline to end of follow-up. The primary safety outcome was participant incidence of at least one trial-defined hypoglycaemic event. Event rates without extractable participant-level numerators and denominators were not converted to participant incidence. Secondary outcomes were change in body weight, achievement of a trial-defined HbA1c target, total daily insulin dose, severe hypoglycaemia requiring external assistance or classified as Level 3, gastrointestinal adverse events, and withdrawals or treatment discontinuations due to adverse events.

### Risk of bias and certainty of evidence

Two reviewers (TM and IM) independently assessed risk of bias using the revised Cochrane risk-of-bias tool for randomised trials (RoB 2) [19]. Result-specific assessments were conducted for HbA1c, trial-defined hypoglycaemia, severe hypoglycaemia, body weight, gastrointestinal adverse events, and withdrawals due to adverse events. Overall assessments were low risk, some concerns, or high risk. Outcomes not reported or not extractable were recorded as not assessable for the review-specific summary, not as a formal RoB 2 category. Protocols and registry records were examined where available for prespecification and potential selective non-reporting. Visualisations were generated using robvis [20].

Certainty of evidence for the same six outcomes was assessed using the Grading of Recommendations Assessment, Development and Evaluation (GRADE) approach [21]. Randomised evidence started at high certainty and was downgraded for risk of bias, inconsistency, indirectness, imprecision, or publication bias. Certainty was classified as high, moderate, low, or very low. For dichotomous outcomes, absolute effects were calculated from the observed comparator-group risk and pooled relative effect.

### Data synthesis and statistical analysis

Meta-analyses were performed using Review Manager 5.4 [22], with the randomised trial as the unit of analysis. For multi-arm trials, one eligible comparator arm was included in each meta-analysis to avoid double-counting. When basal-plus and basal-bolus arms were both eligible, basal-bolus was prioritised because it represented the more fully intensified insulin strategy and most closely matched the review question; otherwise, the extractable eligible basal-plus or premixed arm was used. Where multiple eligible doses of the same intervention were reported as a pooled estimate, that estimate was used. Decisions were documented in the study-to-analysis mapping.

Continuous outcomes were expressed as mean differences (MD) with 95% confidence intervals (95% CI). Standard errors (SEs) were derived from reported standard deviations and group sizes, from the widths of 95% confidence intervals around reported between-group estimates, including estimated treatment differences (ETDs), or from P values using standard methods [23]. Studies without an extractable variance estimate were excluded from the relevant meta-analysis but retained narratively. HbA1c was analysed using a random-effects generic inverse-variance model and body weight using a random-effects inverse-variance model. Dichotomous outcomes were expressed as risk ratios and pooled using Mantel-Haenszel methods. Achievement of a trial-defined HbA1c target and participant incidence of trial-defined hypoglycaemia were analysed using random-effects models. Severe hypoglycaemia was analysed using a fixed-effect model because events were sparse, with risk difference examined in sensitivity analysis.

Clinical heterogeneity was assessed across populations, baseline regimens, interventions, comparators, titration procedures, follow-up, and outcome definitions. Statistical heterogeneity was quantified using I² and Tau-square (τ²) and evaluated using the Chi-square (χ²) test. Prespecified subgroup analyses examined HbA1c by incretin regimen (GLP-1RA plus basal insulin, fixed-ratio/co-formulation, or dual incretin agonist) and clinical pathway (intensification or simplification), and hypoglycaemia by comparator strategy (basal–bolus/basal-plus or premixed/other intensified insulin). HbA1c sensitivity analyses separately excluded IDEAL [27], for which the variance was derived from a P value; SURPASS-6 [15], the tirzepatide trial; DUAL VII, SURPASS-6, and BEGIN VICTOZA ADD-ON [14, 15, 31], for which standard errors were derived from reported confidence intervals; and SIMPLE, Miya et al., and Yamamoto et al. [26, 32, 39], which were small or highly imprecise. Hypoglycaemia sensitivity analyses separately excluded the two very small trials, Miya et al. and Shao et al. [32, 38], and FLAT-SUGAR [30], which used continuous-glucose-monitoring-based ascertainment. Severe hypoglycaemia was additionally analysed using risk difference.

Where pooling was inappropriate because of incompatible definitions, denominators, reporting metrics, or unavailable variance estimates, findings were synthesised narratively in accordance with the Synthesis Without Meta-analysis (SWiM) guideline [24], grouped by intervention and comparator strategy and summarised using direction of effect and key study-level estimates. Small-study effects for HbA1c were assessed by visual inspection of the funnel plot and standard Egger regression of the standard normal deviate on study precision [25]. Formal asymmetry testing was not undertaken for other outcomes because heterogeneity and incompatible reporting limited interpretability, and some outcomes had too few comparable estimates.

## Results

### Study selection and trial characteristics

**Fig. 1:**
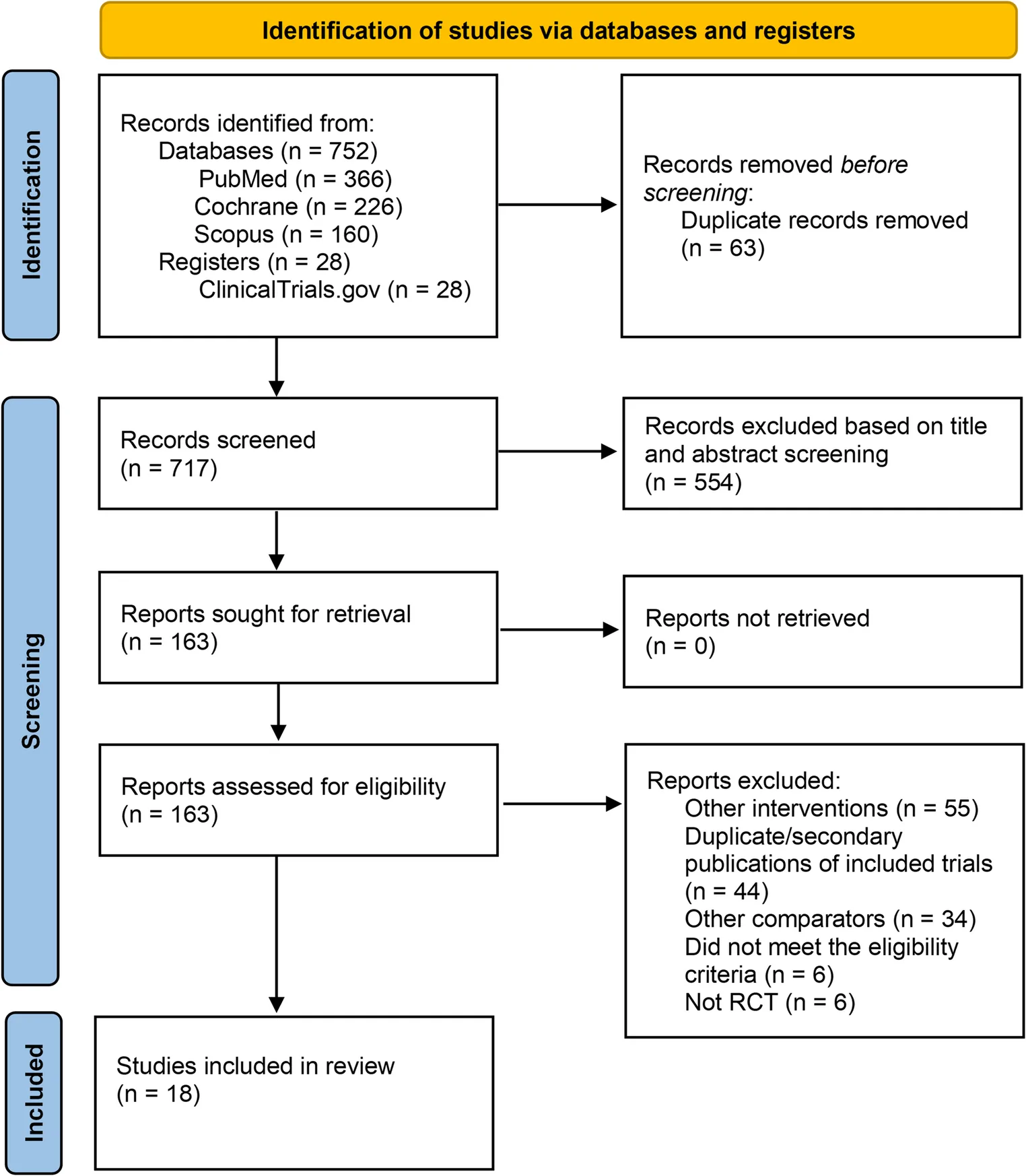
PRISMA flow diagram of the search and selection process for the included articles

The searches identified 780 records: 752 from PubMed/MEDLINE, CENTRAL and Scopus, and 28 from ClinicalTrials.gov. After removal of 63 duplicates, 717 records were screened; 554 were excluded at title/abstract screening and 163 full texts were assessed. Overall, 145 full-text reports were excluded because of ineligible intervention (*n* = 55), duplicate or secondary publication of an included trial (*n* = 44), ineligible comparator (*n* = 34), non-randomised design (*n* = 6), or failure to meet other eligibility criteria (*n* = 6). Eighteen randomised controlled trials were included (Fig. 1).

The 18 included RCTs enrolled adults with T2D requiring injectable intensification or simplification [13–16, 26–39] (Table 1). Most were open-label, parallel-group, multicentre trials [13–15, 27, 28, 30–32, 34–37, 39], several were single-centre or two-centre studies [16, 26, 29, 33, 38], and Yamamoto et al. was additionally a multicentre pilot trial [39]. Mean baseline HbA1c ranged from approximately 6.8% to 12.1%; in 12 trials, it was approximately 7.7–8.8% [13–16, 27, 28, 30, 31, 34–37], whereas SIMPLE and DUAL HIGH enrolled participants with markedly elevated HbA1c [26, 29]. In the vast majority of studies, the mean baseline BMI was ≥ 25 kg/m², indicating predominantly overweight or obese study populations [13–16, 26–39]. Interventions comprised separately administered GLP-1RA plus basal insulin regimens [26–28, 30–33, 35–39], fixed-ratio basal insulin/GLP-1RA combinations or insulin-incretin co-formulations [13, 14, 16, 29, 34], and tirzepatide administered with basal insulin [15]. Comparators comprised basal-bolus insulin [13–16, 26, 28–30, 32, 33, 35–39], basal-plus insulin [31, 36], and premixed or biphasic insulin [27, 34]. Six trials were classified as simplification trials in the clinical-pathway analysis [16, 30, 32, 33, 37, 39]. Follow-up ranged from 12 to 52 weeks and was 26 weeks or six months in 11 trials [14, 16, 26, 29–31, 33–37].

**Table 1:** Characteristics of studies included in the systematic review

Study (trial name), year	Country / setting	Design	Population (key eligibility)	Sample size (randomised / analysed)	Baseline characteristics (mean)	Intervention (incretin-based regimen)	Comparator (insulin strategy)	Follow-up / Endpoint	Key outcomes reported	Hypoglycaemia definition
Billings et al., 2025 COMBINE 3 [13]	Multicentre (14 countries)	RCT; open-label; parallel; non-inferiority	T2D on daily basal insulin; HbA1c 7.0–10.0%.	Ran: 679; Analysed (FAS): 340/339	Age: 60 y; Dur: 14 y; A1c: 8.3%; BMI: 30 kg/m²; Insulin: ~34 U/d	IcoSema (Icodec + Semaglutide) once weekly.	Basal-Bolus; (Glargine U100 + Aspart 2-4x/day).	52 weeks	HbA1c, weight, hypo, insulin dose, PROs.	SMBG < 3.0 mmol/L (< 54 mg/dL) or severe.
Billings et al., 2018 DUAL VII [14]	Multinational (12 countries)	RCT; open-label; parallel; non-inferiority	T2D uncontrolled on Glargine U100 + Metformin (A1c 7–10%).	Ran: 506; Analysed (FAS): 252/254	Age: 58 y; Dur: 13 y; A1c: 8.2%; BMI: 31.7 kg/m²; Insulin: 34 U/d	IDegLira (Fixed-Ratio) OD.; Max dose 50 dose steps.	Basal-Bolus; (Glargine U100 + Aspart 4x/day).	26 weeks	HbA1c, weight, hypo, insulin dose.	Severe or SMBG < 3.1 mmol/L (< 56 mg/dL).
Rosenstock et al., 2023 SURPASS-6 [15]	Multicentre (15 countries)	RCT; open-label; parallel	T2D uncontrolled on basal insulin (A1c 7.5–11%).	Ran: 1428; Analysed (ITT): 716/708	Age: 59 y; Dur: 14 y; A1c: 8.8%; BMI: 33 kg/m²; Insulin: 46 U/d	Tirzepatide QW + Glargine; (5, 10, or 15 mg; pooled analysis).	Basal-Bolus; (Glargine + Lispro TID).	52 weeks	HbA1c, weight, hypo, insulin dose.	SMBG < 54 mg/dL (< 3.0 mmol/L).
Giugliano et al., 2021 BEYOND [16]	Single centre (Italy); outpatient	RCT; open-label; pragmatic; parallel	T2D on full Basal-Bolus (4 injections/day); HbA1c > 7.5%. (Simplification study).	Ran: 305; Analysed: 102/101/102	Age: 62 y; Dur: 17 y; A1c: 8.6%; BMI: 32 kg/m²; Insulin: 53 vs. 49 U/d	Fixed-Ratio Combo (iGlarLixi or IDegLira) OD.; Discontinued bolus insulin.	Basal-Bolus; (Intensified standard care).	6 months (26 weeks)	HbA1c, weight, hypo, insulin dose, DTSQ.	SMBG < 70 mg/dL (Level 1); <54 mg/dL (Level 2).
Abreu et al., 2019 SIMPLE [26]	Single centre (USA); safety-net system	RCT; open-label; parallel; non-inferiority	T2D with very high HbA1c (10%); on any treatment (mostly insulin).	Ran: 120; Analysed (mITT): 54/56	Age: 47 y; Dur: 10 y; A1c: 12.1%; BMI: 37 kg/m²; Insulin: 74 U/d	Liraglutide + Basal (Detemir).; Lira titrated to 1.8 mg.	Basal-Bolus; (Detemir + Aspart).	6 months (26 weeks)	HbA1c, weight, hypo, insulin dose, PROs.	SMBG < 70 mg/dL or severe.
Aso et al., 2021 IDEAL [27]	Multicentre (Japan)	RCT; open-label; parallel	T2D on insulin + OAD; A1c 7.0-10.5%.	Ran: 57; Analysed: 24/28	Age: 68 y; Dur: 20 y; A1c: 7.9% vs. 8.2%; BMI: 25.5 kg/m²; Insulin: ~22 U/d	Insulin degludec plus separately administered liraglutide; liraglutide was titrated to 0.9 mg and insulin degludec was titrated according to protocol.	Premix (IDegAsp) BID.; Co-formulation degludec/aspart.	52 weeks	HbA1c, weight, hypo, insulin dose.	Plasma glucose < 70 mg/dL (< 3.9 mmol/L).
Diamant et al., 2014 4B Study [28]	Multicentre (17 countries); outpatient	RCT; open-label; parallel; non-inferiority	T2D on basal insulin + metformin SU; failed to reach target after 12-week glargine optimisation run-in.	Ran: 637; Analysed (ITT): 315/312	Age: 60 y; Dur: 12 y; A1c: 8.2%; BMI: 32 kg/m²; Insulin: ~61 U/d	Exenatide BID + Insulin Glargine.; Titrated to pre-meal targets.	Basal-Bolus; (Glargine + Lispro TID).	30 weeks	HbA1c, weight, hypo, insulin dose, GI AEs.	SMBG < 3.0 mmol/L (< 54 mg/dL); symptoms or severe.
Galindo et al., 2023 DUAL HIGH [29]	Two centres (USA); academic clinics	RCT; open-label; parallel; non-inferiority	T2D with very high HbA1c (9.0–15.0%); on OADs and/or basal insulin.	Ran: 145; Analysed (ITT): 72/73	Age: 54 y; Dur: NR; A1c: 10.8%; BMI: 32 kg/m²; Insulin: ~0.3 U/kg	IDegLira (Fixed-Ratio) OD.; Titrated to FPG target.	Basal-Bolus; (Degludec + Aspart).	26 weeks	HbA1c, weight, hypo, insulin dose.	SMBG < 70 mg/dL; <54 mg/dL.
Probstfield et al., 2016 FLAT-SUGAR [30]	Multicentre (USA)	RCT; open-label; parallel	T2D on insulin with high CV risk; A1c 6.7-8.0% after run-in on BBI.	Ran: 102; Analysed: 52/50	Age: 62 y; Dur: 15 y; A1c: 7.9%; BMI: 34 kg/m²; Insulin: BBI at run-in	Exenatide (GLIPULIN) + Glargine. Exenatide before meals; stopped rapid insulin.	Basal-Bolus; (Glargine + Rapid-acting insulin).	26 weeks	Glucose variability (CGM), A1c, weight, hypo.	CGM metrics; SMBG < 70 mg/dL.
Mathieu et al., 2014 BEGIN VICTOZA ADD-ON [31]	Multinational (12 countries)	RCT; open-label; parallel	T2D on IDeg + Metformin; HbA1c 7.0–10.0% after 104 weeks of prior IDeg treatment.	Ran: 177; Analysed (FAS): 88/89	Age: 61 y; Dur: ~12 y; A1c: 7.7%; BMI: 32 kg/m²; Insulin: 0.7 U/kg	Liraglutide (up to 1.8 mg) + Insulin Degludec (IDeg).	Basal-Plus; (IDeg + Insulin Aspart OD with largest meal).	26 weeks	HbA1c, weight, hypo, AEs.	SMBG < 3.1 mmol/L (< 56 mg/dL) or severe.
Miya et al., 2018 [32]	Multicentre (Japan)	RCT; open-label; parallel	T2D on MDI (> 3 months); A1c 6.0–9.0%. (Simplification study).	Ran: 31; Analysed: 11/15	Age: 62 y; Dur: 20 y; A1c: 7.2%; BMI: 26.8 kg/m²; Insulin: ~23 U/d	Lixisenatide + Basal Insulin.; Stopped bolus insulin.	Basal-Bolus; (MDI continued).	12 weeks	DTSQ (satisfaction), A1c, weight, hypo.	SMBG < 70 mg/dL or symptoms.
Rodriguez et al., 2024 TRANSITION-T2D [33]	Single-centre (USA)	RCT; open-label; parallel	T2D well-controlled on MDI (A1c 7.5%). (Simplification study).	Ran: 60; Analysed (ITT): 40/20	Age: 69 y; Dur: 18.5 y; A1c: 6.8%; BMI: 35.3 kg/m²; Insulin: 64.5 U/d	Semaglutide QW + Degludec.; Stopped prandial insulin immediately.	Basal-Bolus; (MDI continued).	26 weeks	Maintenance of A1c, weight, insulin dose.	SMBG < 70 mg/dL (Level 1); <54 mg/dL (Level 2).
Rosenstock et al., 2021 SoliMix [34]	Multicentre (17 countries)	RCT; open-label; parallel; non-inferiority	T2D sub optimally controlled on basal insulin + OADs (A1c 7.5–10%).	Ran: 887; Analysed (ITT): 443/444	Age: 60 y; Dur: 13 y; A1c: 8.6%; BMI: 30 kg/m²; Insulin: 34 U/d	iGlarLixi (Fixed-Ratio) OD.; Titrated to fasting target.	Premix (BIAsp 30) BID.; Biphasic insulin aspart.	26 weeks	HbA1c, weight, hypo, insulin dose.	SMBG < 54 mg/dL (Level 2); <70 mg/dL (Level 1).
Rosenstock et al., 2014 Harmony 6 [35]	Multicentre; international	RCT; open-label; parallel; non-inferiority	T2D uncontrolled on glargine (A1c 7-10.5%); 4–8 week glargine standardization run-in.	Ran: 566; Analysed (ITT): 285/281	Age: 55 y; Dur: 11 y; A1c: 8.5%; BMI: ~33 kg/m²; Insulin: ~45 U/d (basal)	Albiglutide QW + Glargine.; Weekly injection.	Basal-Bolus; (Glargine + Lispro TID).	26 weeks (primary)	HbA1c, weight, hypo, GI AEs.	ADA defined; documented < 70 mg/dL.
Rosenstock et al., 2016 GetGoal Duo-2 [36]	Multicentre (18 countries)	RCT; open-label; 3-arm parallel	T2D uncontrolled on basal insulin (A1c 7–9% after optimization).	Ran: 894; Analysed (mITT): 297 (Lixi) / 298(B-Plus) /295 (BB)	Age: 60 y; Dur: 12 y; A1c: 7.8%; BMI: 32 kg/m²; Insulin: ~40 U/d (basal)	Lixisenatide OD + Glargine. Injected before main meal.	Basal-Plus (Glulisine OD) OR Basal-Bolus (Glulisine TID).	26 weeks	HbA1c, weight, hypo, PPG.	Symptomatic with SMBG < 60 mg/dL or severe.
Rosenstock et al., 2020 [37]	Multicentre	RCT; open-label; parallel; non-inferiority	T2D uncontrolled on MDI (basal+prandial). (Simplification study).	Ran: 814; Analysed: 402/412	Age: 58 y; Dur: 15 y; A1c: 7.7%; BMI: 32 kg/m²; Insulin: ~80 U/d	Albiglutide QW + Glargine.; Stopped lispro after 4 weeks.	Basal-Bolus; (Glargine + Lispro TID).	26 weeks	HbA1c, weight, hypo, insulin dose.	Severe or documented < 70 mg/dL.
Shao et al., 2014 [38]	Single centre (China)	RCT; parallel	Newly diagnosed T2D with Obesity and NAFLD.	Ran: 60; Analysed: 30/30	Age: 43 y; Dur: Newly dx; A1c: ~7.6%; BMI: 30.5 kg/m²; Insulin: Naive	Exenatide BID + Glargine.	Basal-Bolus; (Glargine + Aspart TID).	12 weeks	Liver enzymes, liver fat, weight, A1c.	SMBG < 3.9 mmol/L (< 70 mg/dL).
Yamamoto et al., 2018 [39]	Multicentre (Japan)	RCT; open-label; pilot	T2D on MDI; preserved C-peptide. (Simplification study).	Ran: 31; Analysed: 12/13	Age: 61 y; Dur: 10 y; A1c: 7.1%; BMI: 27 kg/m²; Insulin: ~27 U/d	Liraglutide (0.9 mg) + Basal.; Stopped bolus insulin.	Basal-Bolus; (MDI continued).	24 weeks	HbA1c, weight, DTSQ, insulin dose.	SMBG < 70 mg/dL.

Abbreviations used in the table are as follows: *A1c* glycated haemoglobin (HbA1c), *BMI* body mass index, *Dur* duration (specifically diabetes duration in the baseline characteristics column), *n* number of participants, *NR* not reporte, *Ran* randomised (number of participants randomised), *y* years, *FAS* full analysis set, *ITT* intention-to-treat, *mITT* modified intention-to-treat, *RCT* randomised controlled trial, *CV* cardiovascular, *NAFLD* non-alcoholic fatty liver disease, *T2D* type 2 diabetes, *BBI* basal-bolus insulin, *GLP*-*1 RA* glucagon-like peptide-1 receptor agonist, *IcoSema* insulin icodec and semaglutide combination, *IDeg* insulin degludec, *IDegAsp* insulin degludec/insulin aspart co-formulation, *IDegLira* insulin degludec/liraglutide fixed-ratio combination, *iGlarLixi* insulin glargine/lixisenatide fixed-ratio combination, *MDI* multiple daily injections, *OAD* oral antidiabetic drug, *SU* sulphonylurea, *BID* twice daily (bis in die), *OD* once daily, *QW* once weekly, *TID* three times daily, *U* units, *U/d* units per day, *U/kg* units per kilogram, *ADA* American Diabetes Association (specifically referring to their hypoglycaemia classification), *AE* adverse event, *CGM* continuous glucose monitoring, *DTSQ* Diabetes Treatment Satisfaction Questionnaire, *FPG* fasting plasma glucose, *GI* gastrointestinal, *PPG* postprandial glucose, *PRO* patient-reported outcome, *SMBG* self-monitored blood glucose

### Risk of bias

Outcome-specific RoB 2 assessments are summarised in Supplementary Tables S3A-S3F, and HbA1c risk-of-bias visualisations are shown in Supplementary Figs. S1 and S2. For HbA1c, 3/18 trials were judged as low risk and 15/18 raised some concerns; none was at high risk. Concerns were mainly driven by open-label allocation and potential deviations from intended interventions, whereas HbA1c measurement was generally of low risk because it was an objective laboratory outcome. Hypoglycaemia, gastrointestinal adverse events, and withdrawals due to adverse events were more vulnerable to open-label ascertainment, symptom-reporting, and discontinuation-attribution bias. Body weight was objective, but behavioural changes and insulin titration could still be influenced by treatment knowledge. “Not assessable” was used only when a trial did not report, or did not provide extractable data for the review outcome.

### Primary efficacy outcome: change in HbA1c (%)

Seventeen trials contributed to the HbA1c meta-analysis [13–16, 26–37, 39]. One included trial [38] was retained in the systematic review but excluded from the HbA1c meta-analysis because no extractable variance estimate for HbA1c change was available.

**Fig. 2:**
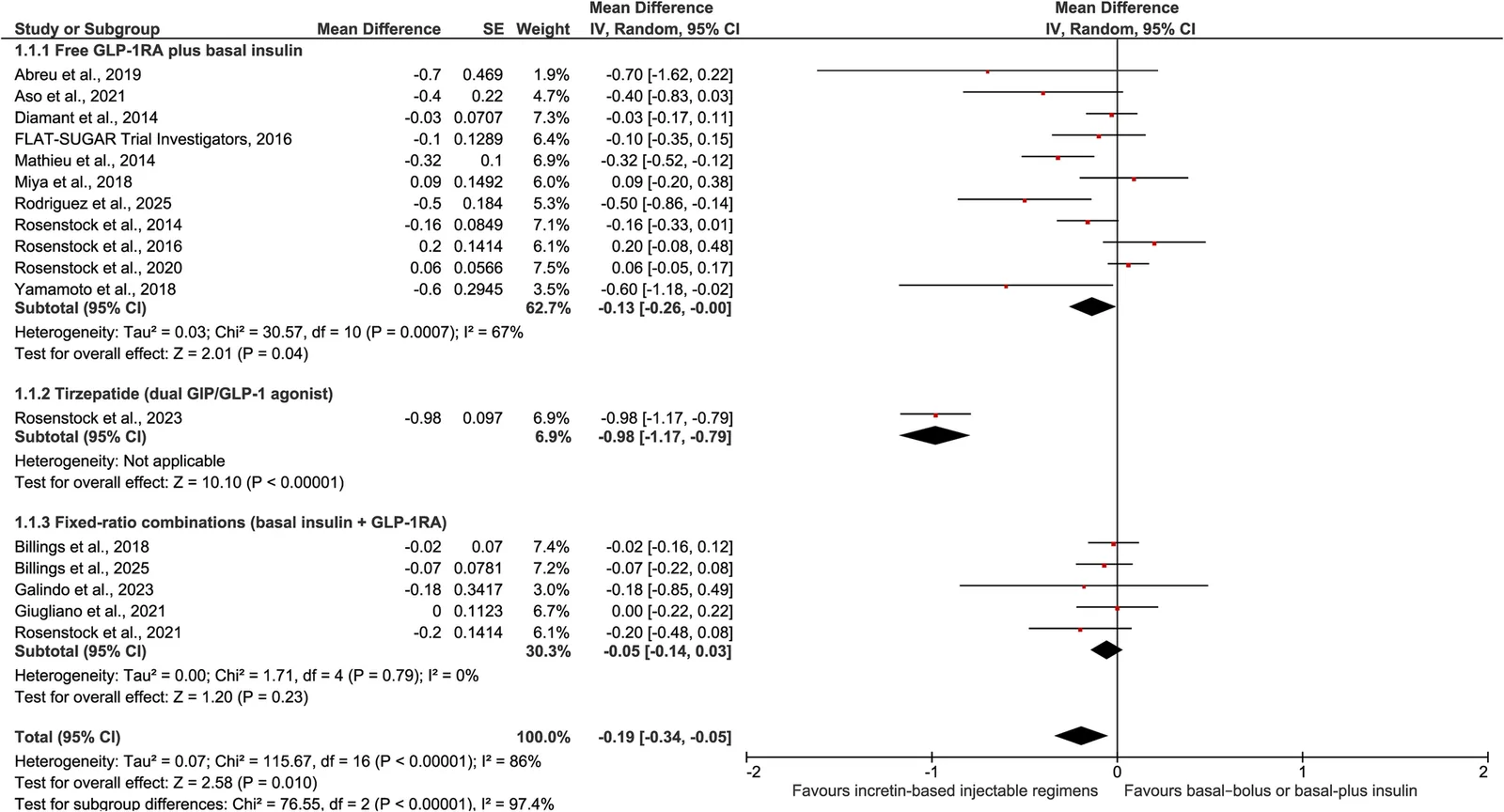
Forest plot of mean differences in HbA1c change from baseline to end of follow-up, comparing incretin-based injectable regimens with intensified insulin strategies. Analyses were performed using a random-effects generic inverse-variance model. Negative mean differences favour incretin-based injectable regimens. Squares represent study-specific effect estimates, horizontal lines represent 95% confidence intervals, and diamonds represent pooled subgroup and overall estimates. Subgroups were defined as separately administered GLP-1RA plus basal insulin, tirzepatide plus basal insulin, and fixed-ratio basal insulin/GLP-1RA combinations or related co-formulations. Included RCTs: [13– [16], 26– [37, 39]

We first analysed the change in HbA1c with incretin-based injectable regimens versus intensified insulin strategies, stratified by incretin regimen (Fig. 2). The overall pooled effect favoured incretin-based regimens (MD -0.19%, 95% CI: -0.34 to -0.05; *P* = 0.01), with substantial heterogeneity (I² = 86%). Effects differed by regimen category (subgroup-difference *P* < 0.00001), highlighting that the overall estimate should be interpreted as an average effect across heterogeneous interventions, comparators, and treatment pathways.

Separately administered GLP-1RA plus basal insulin regimens [26–28, 30–33, 35–37, 39] were associated with a modest but statistically significant reduction in HbA1c versus intensified insulin comparators (MD -0.13%, 95% CI: -0.26 to -0.00; I² = 67%; *P* = 0.04). Tirzepatide plus basal insulin produced the largest HbA1c reduction versus basal-bolus insulin (MD -0.98%, 95% CI: -1.17 to -0.79; *P* < 0.00001) [15]. Fixed-ratio basal insulin/GLP-1RA combinations and related co-formulations [13, 14, 16, 29, 34] showed no statistically significant difference in HbA1c compared with intensified insulin strategies (MD -0.05%, 95% CI: -0.14 to 0.03; I² = 0%; *P* = 0.23).

By clinical pathway, the overall pooled effect favoured incretin-based regimens (MD -0.19%, 95% CI: -0.34 to -0.05; *P* = 0.01), with substantial heterogeneity (I² = 86%) (Supplementary Fig. S3). The outcome of the test for subgroup differences by clinical pathway was not statistically significant (*P* = 0.31). In intensification trials [13–15, 26–29, 31, 34–36], in which participants were inadequately controlled on basal insulin and were randomised to an incretin-based injectable strategy versus prandial, basal-plus, basal-bolus, or premixed insulin intensification, favoured incretin-based treatment (MD -0.23%, 95% CI: -0.43 to -0.03; I² = 89%; *P* = 0.02). In simplification trials [16, 30, 32, 33, 37, 39], where participants already receiving basal-bolus or multiple daily injection therapy were switched to an incretin-based regimen or continued intensified insulin therapy, HbA1c was maintained without a statistically significant between-group difference (MD -0.09%, 95% CI: -0.26 to 0.08; I² = 63%; *P* = 0.29).

Sensitivity analyses generally preserved the direction of effect but showed that statistical significance depended partly on analytical assumptions and influential study classes. Excluding the IDEAL trial [27], for which the variance estimate was derived from the reported P value, the analysis produced a pooled MD of -0.18% (95% CI: -0.33 to -0.03; I² = 87%) maintaining the statistical significance (*P* = 0.02) (Supplementary Fig. S4). Exclusion of the SURPASS-6 trial [15], the pharmacologically distinct tirzepatide trial, attenuated the estimate but retained statistical significance (MD -0.10%, 95% CI: -0.18 to -0.01; I² = 54%; *P* = 0.02) (Supplementary Fig. S5). Excluding trials with SEs derived from 95% confidence intervals around reported ETDs [14, 15, 31] resulted in a non-significant pooled estimate (MD -0.08%, 95% CI: -0.18 to 0.01; I² = 48%; *P* = 0.07) (Supplementary Fig. S6). Finally, when small, imprecise trials [26, 32, 39] were excluded, statistical significance was preserved (MD -0.18%, 95% CI: -0.34 to -0.03; I² = 88%; *P* = 0.02) (Supplementary Fig. S7).

### Primary safety outcome: trial-defined hypoglycaemia

Four trials reported hypoglycaemia only as event rates or without extractable participant incidence and were not included in the participant-incidence meta-analysis [27, 31, 33, 39]. Fourteen trials involving 6,717 participants contributed to the meta-analysis of participants experiencing at least one trial-defined hypoglycaemic event [13–16, 26, 28–30, 32, 34–38].

**Fig. 3:**
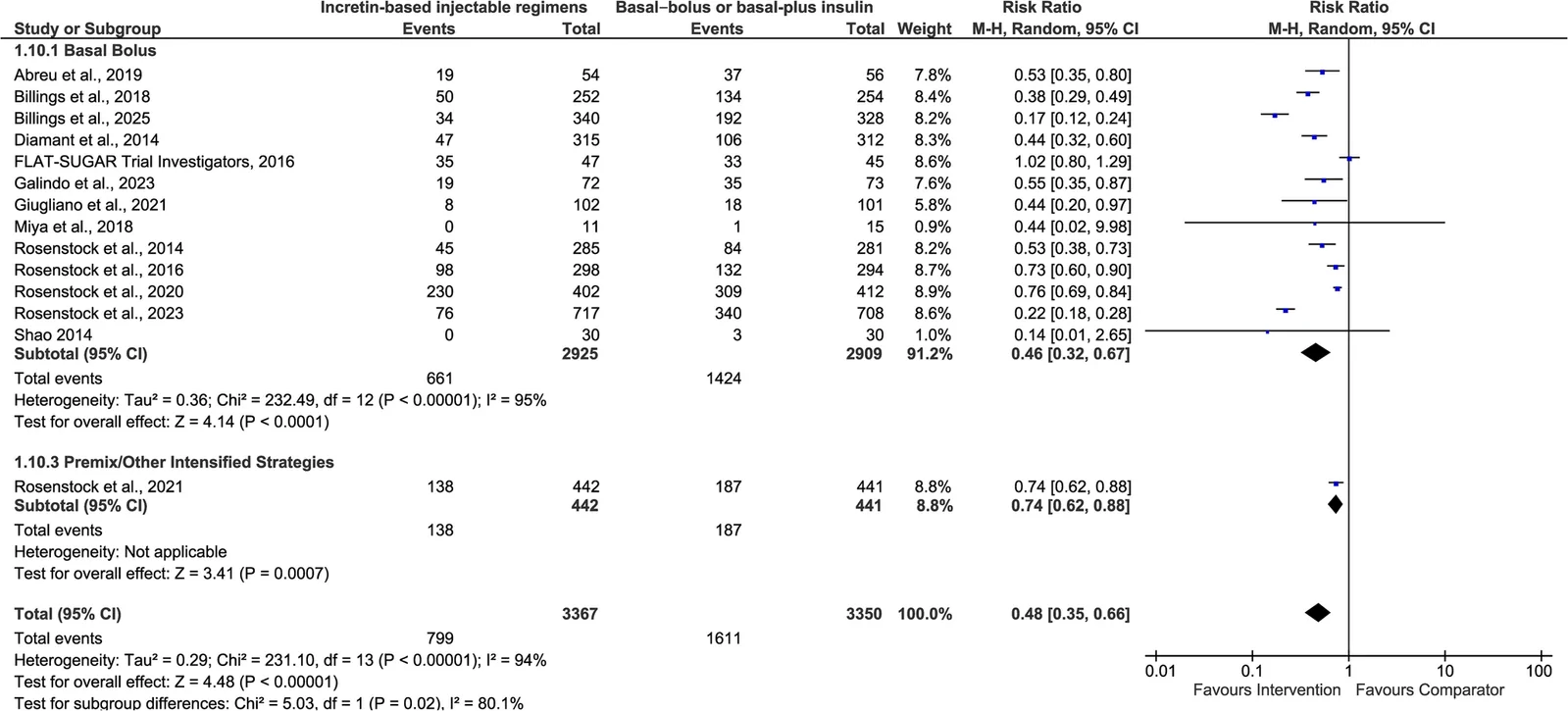
Forest plot of risk ratios for the proportion of participants experiencing at least one trial-defined hypoglycaemic event, comparing incretin-based injectable regimens with intensified insulin strategies. Analyses were performed using a random-effects Mantel-Haenszel model. Risk ratios below 1 favour incretin-based injectable regimens. Squares represent study-specific risk ratios, horizontal lines represent 95% confidence intervals, and diamonds represent pooled subgroup and overall estimates. Comparator subgroups were basal-bolus/basal-plus insulin and premixed or other intensified insulin strategies. Included RCTs: [13– [16, 26], 28– [30, 32], 34– [38]

Hypoglycaemia occurred in 799/3,367 participants receiving incretin-based regimens and 1,611/3,350 receiving intensified insulin. Overall, the analysis showed that incretin-based treatment reduced the risk of trial-defined hypoglycaemia (RR 0.48, 95% CI: 0.35 to 0.66; I² = 94%; *P* < 0.00001), while the subgroup difference was statistically significant (*p* = 0.02) (Fig. 3). In subgroup analysis, trials with basal-bolus or basal-plus comparators showed an RR of 0.46 (95% CI: 0.32 to 0.67; I² = 95%), whereas the single premixed/other intensified-insulin comparison showed an RR of 0.74 (95% CI: 0.62 to 0.88) [34].

Sensitivity analyses were consistent with the primary analysis. Exclusion of the two very small trials, MIYA and Shao et al. [32, 38], produced an RR of 0.49 (95% CI: 0.36 to 0.68; I² = 95%; *P* < 0.0001) (Supplementary Fig. S8). Exclusion of FLAT-SUGAR [30], which used continuous-glucose-monitoring-based ascertainment, produced an RR of 0.45 (95% CI: 0.33 to 0.63; I² = 94%; *P* < 0.00001) (Supplementary Fig. S9).

The four non-poolable trials nevertheless provided relevant rate-based findings. IDEAL reported confirmed hypoglycaemia rates of 0.69 events per patient-year with insulin degludec plus liraglutide and 1.32 events per patient-year with insulin degludec/insulin aspart [27]. BEGIN VICTOZA ADD-ON reported 1.00 versus 8.15 events per patient-year with liraglutide and insulin aspart, respectively, corresponding to an estimated rate ratio of 0.13 (95% CI: 0.08 to 0.21) [31]. TRANSITION-T2D reported estimated mean event rates of 0.7 with semaglutide-based simplification and 1.4 with continued multiple daily injections, with a rate ratio of 2.1 (95% CI: 0.6 to 7.5) for continued multiple daily injections versus semaglutide-based simplification [33]. Yamamoto et al. (2018) [39] reported a numerically lower frequency of hypoglycaemia with liraglutide plus basal insulin, but participant-incidence data were not extractable.

### Secondary efficacy outcomes

**Fig. 4:**
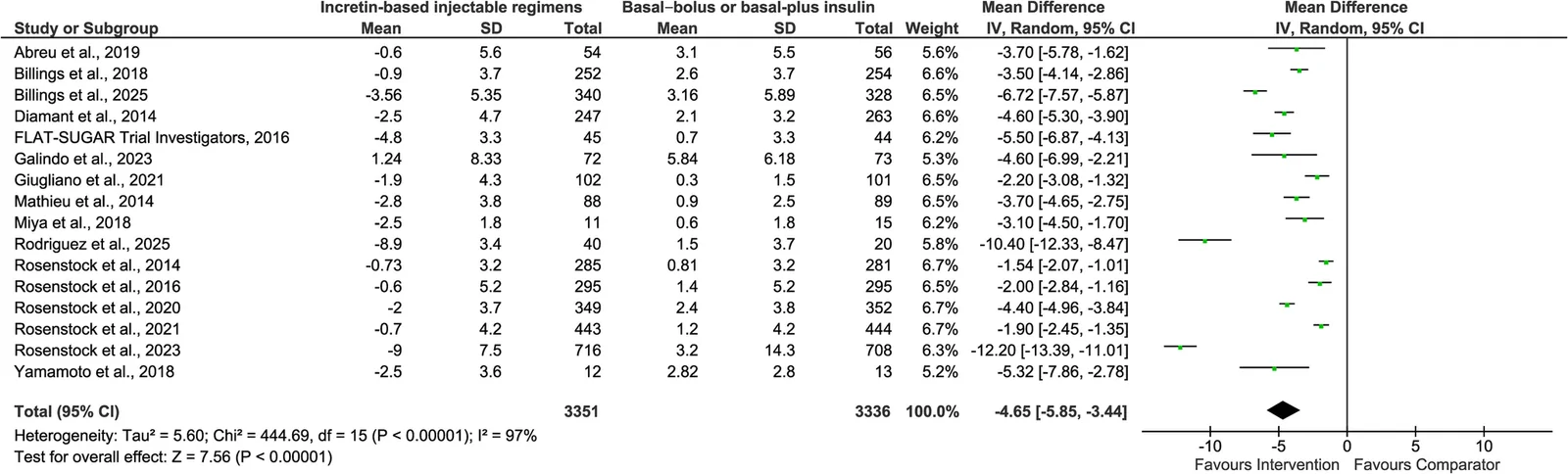
Forest plot of mean differences in body-weight change from baseline to end of follow-up, comparing incretin-based injectable regimens with intensified insulin strategies. Analyses were performed using a random-effects inverse-variance model. Negative mean differences favour incretin-based injectable regimens. Squares represent study-specific effect estimates, horizontal lines represent 95% confidence intervals, and the diamond represents the pooled overall estimate. Included RCTs: [13– [16, 26], 28– [37, 39]

Sixteen trials (*n* = 6,687) contributed to body-weight meta-analysis. Incretin-based regimens produced greater weight reduction than intensified insulin (MD -4.65 kg, 95% CI: -5.85 to -3.44; I² = 97%; *P* < 0.00001) (Fig. 4), with very high heterogeneity reflecting drug potency and regimen type [13–16, 26, 28–37, 39]. Study-level body-weight data are provided in Supplementary Table S4.

Twelve trials (*n* = 6,515) reported HbA1c target achievement, usually HbA1c < 7.0%; incretin-based regimens increased target attainment (RR 1.27, 95% CI: 1.05 to 1.54; I² = 92%; *P* = 0.01) (Supplementary Fig. S10) [13–16, 26–28, 31, 34–37]. Study-level HbA1c target-achievement data are provided in Supplementary Table S5.

Total daily insulin dose was not meta-analysed because dose metrics were heterogeneous, but narrative synthesis showed lower insulin exposure with incretin-based regimens; endpoint doses were lower in DUAL VII [14] (40 vs. 84 units/day), the 4B Study [28] (56.9 vs. 93.7 units/day), and SIMPLE [26] (59.4 vs. 94.5 units/day), and BEYOND showed a reduction with simplification (-27.1 vs. + 12.3 units/day) [16]. Study-level insulin-dose data and the SWiM-style narrative synthesis are provided in Supplementary Table S6.

### Secondary safety and tolerability outcomes

Seven trials with at least one severe hypoglycaemic event across the comparison groups contributed estimable information to the risk-ratio meta-analysis (*n* = 4,715) [13–15, 26, 28, 35, 37]. Severe hypoglycaemia occurred in 20/2,363 participants receiving incretin-based regimens and 66/2,352 receiving intensified insulin, favouring incretin-based treatment (RR 0.32, 95% CI: 0.19 to 0.51; I² = 29%; *P* < 0.00001) (Supplementary Fig. S11). Additional trials reported no severe events in either group or did not provide extractable participant-incidence data. The risk-difference sensitivity analysis was consistent with the primary analysis (RD -0.02, 95% CI: -0.03 to -0.01; I² = 74%; *P* < 0.00001) (Supplementary Fig. S12). Study-level severe-hypoglycaemia data are provided in Supplementary Table S7.

Gastrointestinal adverse events were not pooled because symptom definitions, ascertainment, and participant-level reporting differed substantially. Where symptom-specific data were available, nausea was more frequent with incretin-based regimens, including SIMPLE (32/54 vs. 16/56) [26], COMBINE 3 (74/340 vs. 8/328) [13], Harmony 6 (32/285 vs. 4/281) [35], and SURPASS-6 (143/717 vs. 8/708) [15]. In SURPASS-6, vomiting (62/717 vs. 4/708) and diarrhoea (91/717 vs. 17/708) showed the same direction [15]. Study-level gastrointestinal adverse-event data and reporting notes are provided in Supplementary Table S8.

Withdrawals due to adverse events were generally infrequent but more common in several incretin arms, including SURPASS-6 (43/717 vs. 17/708) [15], Harmony 6 (15/285 vs. 1/281) [35], and GetGoal Duo-2 (15/298 vs. 3/294) [36]. In other studies, withdrawals were low or similar between groups, including SoliMix (3/442 vs. 3/441) [34]. Study-level withdrawals due to adverse-event data are provided in Supplementary Table S9.

### Small-study effects and certainty of evidence

**Fig. 5:**
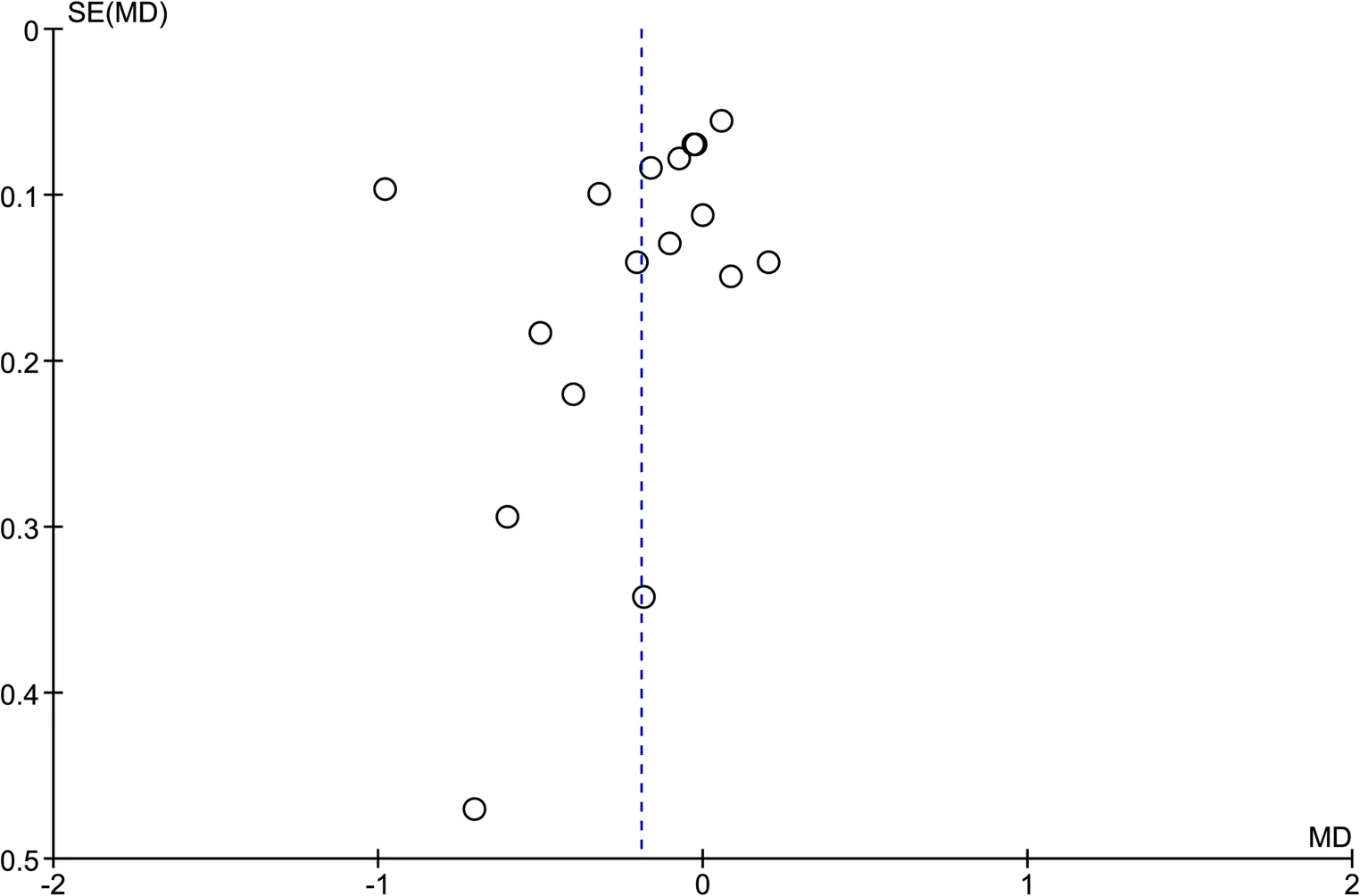
Funnel plot assessing small-study effects for the meta-analysis of change in HbA1c (%). The x-axis shows the mean difference (MD), and the y-axis shows the standard error of the mean difference [SE(MD)]. Each circle represents one study estimate, and the dashed vertical line indicates the pooled effect estimate. Standard Egger regression did not provide clear evidence of funnel-plot asymmetry (intercept − 1.82, 95% CI: -4.88 to 1.24; *P* = 0.224) [13– [16], 26– [37, 39]

For HbA1c, visual inspection of the funnel plot did not indicate clear asymmetry (Fig. 5). Standard Egger regression did not provide evidence of funnel-plot asymmetry (intercept − 1.82, 95% CI: -4.88 to 1.24; *p* = 0.224), although interpretation was limited by substantial between-study heterogeneity [25]. GRADE findings are summarised in Supplementary Table S10, with the detailed downgrading rationale provided in Supplementary Table S11. Certainty was low for HbA1c, trial-defined hypoglycaemia, body weight, and withdrawals due to adverse events; moderate for severe hypoglycaemia; and very low for gastrointestinal adverse events.

## Discussion

This systematic review and meta-analysis synthesised evidence collected from 18 RCTs, assessing whether, in adults with T2D requiring injectable treatment escalation or simplification, incretin-based injectable regimens offer advantages over intensified insulin approaches in terms of glycaemic control along with safety and tolerability. Overall, we found that incretin-based injectable strategies showed a favourable profile compared with intensified insulin regimens. Incretin-based regimens produced a small average HbA1c advantage and increased HbA1c target attainment while more consistent benefits were observed for trial-defined hypoglycaemia, severe hypoglycaemia, body weight, and insulin exposure. Gastrointestinal adverse events were more frequent with incretin-based therapy, and withdrawals due to adverse events were higher in several trials but generally uncommon.

These findings require cautious interpretation. Heterogeneity was substantial for HbA1c, hypoglycaemia, and body weight, and GRADE certainty was low for HbA1c, trial-defined hypoglycaemia, body weight, and adverse-event withdrawals; moderate for severe hypoglycaemia; and very low for gastrointestinal adverse events. The pooled estimates therefore represent average effects across clinically distinct regimens and pathways rather than a uniform class effect.

The findings are directionally consistent with earlier systematic reviews while extending their clinical scope. Eng et al. [40] broadly evaluated GLP-1RA plus basal insulin across several antidiabetic comparators, whereas Liakopoulou et al. [41] focused on fixed-ratio basal insulin/GLP-1RA combinations. Maiorino et al. [42] examined separately administered and fixed-ratio basal insulin/GLP-1RA regimens versus basal-insulin intensification but did not centre the question exclusively on intensified-insulin comparators or distinguish intensification from simplification. Castellana et al. [12] provided the closest prior synthesis, comparing GLP-1RA/insulin combinations with basal-plus or basal-bolus insulin and reporting similar glycaemic efficacy with advantages for weight, hypoglycaemia, and insulin dose. The present review incorporates subsequent simplification trials, tirzepatide/SURPASS-6, IcoSema/COMBINE 3, and a premixed-insulin comparator, while analysing intensification and simplification as clinically distinct pathways [13, 15, 16, 34].

The largest HbA1c separation was observed with tirzepatide plus basal insulin in SURPASS-6 [15]. However, this subgroup comprised a single trial and should not be interpreted as a direct comparative ranking of incretin strategies or as a class-wide dual-agonist effect. Separately administered GLP-1RAs plus basal insulin produced a smaller average HbA1c advantage, indicating that superiority over prandial escalation is not uniform across agents, populations, or titration protocols [12, 40]. Fixed-ratio combinations and insulin-incretin co-formulations showed no statistically significant pooled HbA1c difference from intensified insulin. This supports comparable glycaemic efficacy but does not constitute a meta-analytic demonstration of non-inferiority, which remains a trial-specific conclusion. The extreme heterogeneity in body-weight effects similarly indicates that the pooled reduction was strongly influenced by regimen potency, particularly tirzepatide, and should not be applied uniformly to all included strategies.

Clinical pathway also modifies interpretation. Intensification trials showed a modest HbA1c advantage, whereas simplification trials maintained HbA1c without a statistically significant between-group difference. Although the formal test for subgroup interaction was not significant, separating these pathways remains clinically informative because their treatment objectives differ: intensification seeks additional glycaemic lowering after basal-insulin failure, whereas simplification seeks to reduce treatment burden while preserving control in people already receiving basal-bolus or multiple-daily-injection regimens. In several simplification trials, preserved HbA1c was accompanied by lower insulin exposure, fewer injections, less hypoglycaemia, or weight benefit; these outcomes should be interpreted at trial level because they were not uniformly pooled [16, 32, 33, 37, 39].

The reduction in hypoglycaemia is clinically important, but the pooled estimate showed marked heterogeneity (I²=94%). Trials used different biochemical thresholds, symptom-based definitions, monitoring methods, and reporting metrics, including participant incidence and event rates. The consistent direction of sensitivity analyses supports the robustness of the safety signal, but the precise magnitude should not be assumed across all regimens. Severe hypoglycaemia was also reduced, although events were sparse and estimates remain sensitive to rare-event methods [43, 44].

Gastrointestinal tolerability was the principal disadvantage of incretin-based therapy. Nausea, vomiting, and diarrhoea were more frequent in several incretin arms, and discontinuations were higher in SURPASS-6, Harmony 6, and GetGoal Duo-2 [15, 35, 36]. Because symptom definitions, ascertainment, and denominators differed, gastrointestinal outcomes could not be pooled and their comparative magnitude remains uncertain. Net benefit should therefore be considered as a balance among glycaemic efficacy, hypoglycaemia, weight, treatment burden, and tolerability rather than as unconditional superiority [45, 46].

For adults inadequately controlled on basal insulin, the findings support considering an incretin-based injectable strategy before routine progression to basal-bolus insulin when hypoglycaemia avoidance, weight management, lower insulin exposure, or injection burden are priorities. The evidence also supports simplification in carefully selected people receiving multiple daily injections when glycaemic control can be monitored during prandial-insulin withdrawal. Prandial insulin remains appropriate in catabolic states, marked insulin deficiency, incretin intolerance or contraindication, and circumstances requiring flexible mealtime correction. Treatment selection should also incorporate access, cost, patient preference, gastrointestinal tolerability, and capacity for titration and follow-up, none of which was comprehensively evaluated in the included trials [3, 7].

The strengths of our systematic review include the clinically focused comparison with intensified-insulin strategies as well as the inclusion of both intensification and simplification pathways. Furthermore, we applied a comprehensive search strategy and focused on outcomes that are clinically relevant. Prespecified subgroup and sensitivity analyses and retention of relevant non-poolable data through narrative synthesis enhance the validity of our analysis. Study-to-report and study-to-analysis mapping also reduced the risk of double-counting secondary publications and participants from multi-arm trials.

Some limitations should be considered when interpreting our findings. Most trials were open-label, increasing susceptibility to bias in symptom reporting, hypoglycaemia ascertainment, treatment deviations, and discontinuation decisions, although objective outcomes such as HbA1c and measured body weight were less vulnerable [47, 48]. Heterogeneity was substantial for HbA1c, hypoglycaemia, and body weight; pooled estimates therefore represent average effects across clinically heterogeneous strategies, not uniform class effects. Certainty was low for several outcomes and very low for gastrointestinal adverse events [21]. Follow-up was generally 12–52 weeks, limiting inference about durability, persistence, and rare or long-term harms. Generalisability is also constrained by trial eligibility criteria and the predominance of structured treat-to-target protocols.

Future trials should use standardised hypoglycaemia definitions and report both participant incidence and event rates. Simplification studies should prespecify prandial-insulin withdrawal and rescue algorithms and consistently assess injection burden, treatment satisfaction, insulin dose, adherence, persistence, and cost. Longer pragmatic trials are needed to determine durability, rare adverse events, and effectiveness across broader clinical populations, with direct comparisons among contemporary incretin-containing strategies where clinically justified [44].

## Conclusion

Incretin-based injectable regimens may provide a favourable alternative to intensified insulin for selected adults with type 2 diabetes. Their most consistent advantages were lower hypoglycaemia risk, fewer severe events, lower body weight, and reduced insulin exposure, whereas the average HbA1c advantage was small and heterogeneous. Simplification generally preserved glycaemic control rather than producing superior HbA1c lowering. These benefits should be balanced against gastrointestinal tolerability and interpreted by regimen, clinical pathway, and certainty of evidence.

## Supplementary Information

Below is the link to the electronic supplementary material.

Supplementary Material 1

## Data Availability

All data analysed in this review were derived from published reports, supplementary materials, and trial-register records. Extracted study-level data, risk-of-bias judgements, GRADE assessment, search strategies, and supplementary analyses are provided in the supplementary material. All data are available from the corresponding author upon reasonable request.
