# Supplementary material for: Incretin-based injectable strategies versus intensified insulin for treatment intensification and simplification in type 2 diabetes: a systematic review and meta-analysis
Source: J Diabetes Metab Disord. 2026 Sep 26;25(2):270. doi: 10.1007/s40200-026-02089-x (PMC13615951; doi:10.1007/s40200-026-02089-x)
Supplement: Supplementary file 1 [file 40200_2026_2089_MOESM1_ESM.docx]

**Table S1: Database specific search strings and relative keywords.**

| **Database** | **Search Strategy** | **Filters/Results** |
| --- | --- | --- |
| PubMed | **Query:** ( "Diabetes Mellitus, Type 2"[Mesh] OR "type 2 diabetes"[tiab] OR T2DM[tiab] OR T2D[tiab] ) AND ( "glucagon-like peptide-1 receptor agonist*"[tiab] OR "GLP-1 receptor agonist*"[tiab] OR "GLP-1RA"[tiab] OR "GLP-1 RA"[tiab] OR liraglutide[tiab] OR semaglutide[tiab] OR dulaglutide[tiab] OR exenatide[tiab] OR lixisenatide[tiab] OR albiglutide[tiab] OR IDegLira[tiab] OR iDegLira[tiab] OR iGlarLixi[tiab] OR tirzepatide[tiab] OR "GIP/GLP-1"[tiab] ) AND ( "insulin glargine"[tiab] OR "insulin degludec"[tiab] OR "insulin detemir"[tiab] OR "insulin aspart"[tiab] OR "insulin lispro"[tiab] OR "insulin glulisine"[tiab] OR "biphasic insulin aspart"[tiab] OR "BIAsp 30"[tiab] OR premix*[tiab] OR "basal-bolus"[tiab] OR "basal bolus"[tiab] OR "basal-plus"[tiab] OR "basal plus"[tiab] OR "prandial insulin"[tiab] OR "mealtime insulin"[tiab] OR "multiple daily injection*"[tiab] OR MDI[tiab] OR "intensive insulin therap*"[tiab] ) AND ( "Randomized Controlled Trial"[pt] OR "Clinical Trial"[pt] OR random*[tiab] ) | No additional interface filters were applied; limits were embedded in the search strategy  **Results:** 366 |
| Cochrane | **Query:** (type 2 diabet* OR T2DM OR T2D) AND ("glucagon-like peptide-1" OR "glucagon like peptide-1" OR "GLP-1" OR "GLP1" OR "GLP-1 receptor agonist*" OR liraglutide OR semaglutide OR dulaglutide OR exenatide OR lixisenatide OR albiglutide OR IDegLira OR iDegLira OR iGlarLixi OR tirzepatide OR "GIP/GLP-1" OR "GIP GLP-1") AND ("basal-bolus" OR "basal bolus" OR "basal-plus" OR "basal plus" OR "multiple daily injection*" OR MDI OR premix* OR "biphasic insulin" OR "BIAsp 30" OR "prandial insulin" OR "mealtime insulin" OR "intensive insulin therap*") | No additional limits applied (searched CENTRAL via Cochrane Library)  **Results:** 226 |
| ClinicalTrials.gov | **Query:** (liraglutide OR semaglutide OR dulaglutide OR exenatide OR lixisenatide OR albiglutide OR IDegLira OR iGlarLixi OR tirzepatide OR IcoSema) AND ("basal-bolus" OR "basal bolus" OR "basal-plus" OR "basal plus" OR "multiple daily injections" OR MDI OR premix OR "biphasic insulin" OR "BIAsp 30" OR "prandial insulin" OR "mealtime insulin") | **Filters:** Adults with type 2 diabetes mellitus, phases 2-4, with study status “Completed”, “Terminated” or “Active, not recruiting”.  **Results:** 28 |
| Scopus | **Query:** ( TITLE-ABS-KEY ( "type 2 diabet*" OR t2dm OR t2d ) AND TITLE-ABS-KEY ( "glucagon-like peptide-1" OR "glucagon like peptide-1" OR "GLP-1" OR "GLP1" OR "GLP-1 receptor agonist*" OR liraglutide OR semaglutide OR dulaglutide OR exenatide OR lixisenatide OR albiglutide OR "IDegLira" OR ideglira OR "iDegLira" OR "iGlarLixi" OR tirzepatide OR "IcoSema" OR "GIP/GLP-1" OR "GIP GLP-1" ) AND TITLE-ABS-KEY ( "basal-bolus" OR "basal bolus" OR "basal-plus" OR "basal plus" OR "multiple daily injection*" OR mdi OR premix* OR "biphasic insulin" OR "BIAsp 30" OR "prandial insulin" OR "mealtime insulin" OR "intensive insulin therap*" ) AND TITLE-ABS-KEY ( random* ) ) AND NOT TITLE-ABS-KEY ( "type 1 diabet*" OR "juvenile diabet*" OR "gestational diabet*" ) AND ( LIMIT-TO ( DOCTYPE , "ar" ) ) AND ( LIMIT-TO ( EXACTKEYWORD , "Human" ) ) AND ( LIMIT-TO ( LANGUAGE , "English" ) ). | **Filters:** Limited to Article, Humans, English  **Results:** 160 |

**Table S2. Study-to-report and study-to-analysis mapping**

QC summary: 18 included primary reports; 44 linked duplicate/secondary reports; 62 total linked reports. Pooled analyses: HbA1c n=17; trial-defined hypoglycaemia n=14; severe hypoglycaemia RR n=7; body weight n=16; HbA1c target achievement n=12. Dose, gastrointestinal adverse events, and withdrawals due to adverse events were retained for narrative/SWiM synthesis only.

| **Study / trial** | **Reports Primary; secondary** | **Path** | **Incretin strategy** | **Selected comparator / analysis decision** | **Pooled outcomes included** | **Key non-poolability / notes** |
| --- | --- | --- | --- | --- | --- | --- |
| Diamant et al., 2014  4B Study [28] | 43; sec n=4 (9; 42; 123; 124) | I | Free GLP-1RA + basal insulin | Basal-bolus glargine + lispro TID | A1c, Hypo, Sev, Wt, Tgt | — |
| Mathieu et al., 2014  BEGIN VICTOZA ADD-ON [31] | 21; sec n=0 | I | Free GLP-1RA + basal insulin | Basal-plus IDeg + aspart OD | A1c, Wt, Tgt | Hypo non-poolable. Sev RR not estimable: 0 events. |
| Giugliano et al., 2021  BEYOND [16] | 131; sec n=1 (7) | S | FRC basal insulin/GLP-1RA | Basal-bolus standard care | A1c, Hypo, Wt, Tgt | FRC arm selected; basal insulin+SGLT2i arm excluded. Sev incidence not extractable. |
| Billings et al., 2025  COMBINE 3 [13] | 1; sec n=2 (34; 119) | I | Once-weekly IcoSema | Basal-bolus glargine + aspart 2-4×/day | A1c, Hypo, Sev, Wt, Tgt | — |
| Galindo et al., 2023  DUAL HIGH [29] | 6; sec n=3 (53; 54; 133) | I | FRC basal insulin/GLP-1RA | Basal-bolus degludec + aspart | A1c, Hypo, Wt | Sev RR not estimable: 0 events. Tgt unavailable. |
| Billings et al., 2018  DUAL VII [14] | 36; sec n=5 (5; 33; 35; 37; 118) | I | FRC basal insulin/GLP-1RA | Basal-bolus glargine + aspart 4×/day | A1c, Hypo, Sev, Wt, Tgt | — |
| Probstfield et al., 2016  FLAT-SUGAR [30] | 61; sec n=2 (137; 138) | S | Free GLP-1RA + basal insulin | Basal-bolus glargine + rapid-acting insulin | A1c, Hypo, Wt | Sev RR not estimable: 0 events. Tgt unavailable. |
| GetGoal Duo-2 (Rosenstock et al., 2016) | 28; sec n=1 (105) | I | Free GLP-1RA + basal insulin | Basal-plus (Glulisine OD) OR Basal-bolus (Glulisine TID) | A1c, Hypo, Wt, Tgt | BB glulisine TID selected; basal-plus excluded. Sev RR not estimable: 0 events. |
| Rosenstock et al., 2014  Harmony 6 [35] | 26; sec n=1 (111) | I | Free GLP-1RA + basal insulin | Basal-bolus glargine + lispro TID | A1c, Hypo, Sev, Wt, Tgt | — |
| Aso et al., 2021  IDEAL [27] | 4; sec n=0 | I | Free GLP-1RA + basal insulin | Premix (IDegAsp) BID. Co-formulation degludec/aspart | A1c, Tgt | Hypo non-poolable. Sev incidence not extractable. Wt variance unavailable. |
| Miya et al., 2018 [32] | 16; sec n=1 (88) | S | Free GLP-1RA + basal insulin | Basal-bolus MDI continued | A1c, Hypo, Wt | Sev RR not estimable: 0 events. Tgt unavailable. |
| Rodriguez et al., 2024  TRANSITION-T2D [33] | 25; sec n=3 (100; 101; 139) | S | Free GLP-1RA + basal insulin | Basal-bolus MDI continued | A1c, Wt | Hypo non-poolable. Severe hypo not pooled. Tgt unavailable. |
| Rosenstock et al., 2020 [37] | 106; sec n=2 (107; 154) | S | Free GLP-1RA + basal insulin | Basal-bolus glargine + lispro TID | A1c, Hypo, Sev, Wt, Tgt | — |
| Abreu et al., 2019  SIMPLE [26] | 3; sec n=1 (117) | I | Free GLP-1RA + basal insulin | Basal-bolus detemir + aspart | A1c, Hypo, Sev, Wt, Tgt | — |
| Shao et al., 2014 | 129; sec n=0 | I | Free GLP-1RA + basal insulin | Basal-bolus glargine + aspart TID | Hypo | A1c variance unavailable. Severe hypo not pooled. Wt variance unavailable. Tgt unavailable. |
| Rosenstock et al., 2021  SoliMix [34] | 103; sec n=16 (10; 22; 40; 50; 51; 62; 81; 82; 83; 97; 98; 102; 109; 130; 136; 145) | I | FRC basal insulin/GLP-1RA | Premix BIAsp 30 BID Biphasic insulin aspart | A1c, Hypo, Wt, Tgt | Severe hypo not pooled. |
| Rosenstock et al., 2023  SURPASS-6 [15] | 27; sec n=1 (125) | I | Tirzepatide + basal insulin | Basal-bolus glargine + lispro TID | A1c, Hypo, Sev, Wt, Tgt | Pooled tirzepatide doses used. |
| Yamamoto et al., 2018 | 93; sec n=1 (115) | S | Free GLP-1RA + basal insulin | Basal-bolus MDI continued | A1c, Wt | Hypo non-poolable. Sev RR not estimable: 0 events. Tgt unavailable. |

**Abbreviations:** **I**, intensification; **S**, simplification; **A1c**, change in HbA1c meta-analysis; **Hypo**, trial-defined hypoglycaemia participant-incidence meta-analysis; **Sev,** severe hypoglycaemia risk-ratio meta-analysis; **Wt**, body-weight meta-analysis; **Tgt,** HbA1c target-achievement meta-analysis; **FRC**, fixed-ratio combination; BB, basal-bolus.

**Supplementary Table S3. Outcome-specific RoB 2 assessments for included randomised trials**

**Table S3A. Outcome-specific RoB 2 assessment for change in HbA1c**

| **Study** | **D1** | **D2** | **D3** | **D4** | **D5** | **Overall** | **Concise rationale** |
| --- | --- | --- | --- | --- | --- | --- | --- |
| Billings et al., 2025  COMBINE 3 [13] | Low | Low | Low | Low | Low | Low | Adequate randomisation/analysis; objective laboratory outcome. |
| Billings et al., 2018  DUAL VII [14] | Low | Some concerns | Low | Low | Low | Some concerns | Open-label design; objective hba1c, but treatment/titration deviations possible. |
| Rosenstock et al., 2023  SURPASS-6 [15] | Low | Low | Low | Low | Low | Low | Adequate randomisation/analysis; objective laboratory outcome. |
| Giugliano et al., 2021  BEYOND [16] | Low | Some concerns | Low | Low | Low | Some concerns | Open-label design; objective hba1c, but treatment/titration deviations possible. |
| Abreu et al., 2019  SIMPLE [26] | Low | Some concerns | Some concerns | Low | Low | Some concerns | Open-label design; objective hba1c, but treatment/titration deviations possible; missing-data/attrition concern. |
| Aso et al., 2021  IDEAL [27] | Some concerns | Some concerns | Low | Low | Low | Some concerns | Open-label design; objective hba1c, but treatment/titration deviations possible; allocation/randomisation detail unclear. |
| Diamant et al., 2014  4B Study [28] | Low | Some concerns | Low | Low | Low | Some concerns | Open-label design; objective hba1c, but treatment/titration deviations possible. |
| Galindo et al., 2023  DUAL HIGH [29] | Low | Some concerns | Low | Low | Low | Some concerns | Open-label design; objective hba1c, but treatment/titration deviations possible. |
| Probstfield et al., 2016  FLAT-SUGAR [30] | Low | Some concerns | Low | Low | Low | Some concerns | Open-label design; objective hba1c, but treatment/titration deviations possible. |
| Mathieu et al., 2014  BEGIN VICTOZA ADD-ON [31] | Low | Some concerns | Low | Low | Low | Some concerns | Open-label design; objective hba1c, but treatment/titration deviations possible. |
| Miya et al., 2018 [32] | Low | Some concerns | Low | Low | Low | Some concerns | Open-label design; objective hba1c, but treatment/titration deviations possible. |
| Rodriguez et al., 2024  TRANSITION-T2D [33] | Low | Some concerns | Low | Low | Low | Some concerns | Open-label design; objective hba1c, but treatment/titration deviations possible. |
| Rosenstock et al., 2021  SoliMix [34] | Low | Low | Low | Low | Low | Low | Adequate randomisation/analysis; objective laboratory outcome. |
| Rosenstock et al., 2014  Harmony 6 [35] | Low | Some concerns | Low | Low | Low | Some concerns | Open-label design; objective hba1c, but treatment/titration deviations possible. |
| Rosenstock et al., 2016  GetGoal Duo-2 [36] | Low | Some concerns | Low | Low | Low | Some concerns | Open-label design; objective hba1c, but treatment/titration deviations possible. |
| Rosenstock et al., 2020 [37] | Low | Some concerns | Low | Low | Low | Some concerns | Open-label design; objective hba1c, but treatment/titration deviations possible. |
| Shao et al., 2014 [38] | Some concerns | Some concerns | Low | Low | Some concerns | Some concerns | Open-label design; objective hba1c, but treatment/titration deviations possible; allocation/randomisation detail unclear; protocol/registry or reporting detail limited. |
| Yamamoto et al., 2018 [39] | Low | Some concerns | Low | Low | Low | Some concerns | Open-label design; objective hba1c, but treatment/titration deviations possible. |

D1, randomisation process; D2, deviations from intended interventions; D3, missing outcome data; D4, measurement of the outcome; D5, selection of the reported result. “Not assessable” indicates that no usable review-specific result was available and is not a formal RoB 2 domain judgement.

*Panel note: Objective outcomes: D4 was judged low unless problematic measurement procedures were identified; open-label designs mainly informed D2 and possible treatment/titration deviations.*

**Table S3B. Outcome-specific RoB 2 assessment for change in body weight**

| **Study** | **D1** | **D2** | **D3** | **D4** | **D5** | **Overall** | **Concise rationale** |
| --- | --- | --- | --- | --- | --- | --- | --- |
| Billings et al., 2025  COMBINE 3 [13] | Low | Low | Low | Low | Low | Low | Objective endpoint; prespecified/complete reporting with low missing-data concern. |
| Billings et al., 2018  DUAL VII [14] | Low | Some concerns | Low | Low | Low | Some concerns | Open-label; objective weight, but behavioural/titration effects possible. |
| Rosenstock et al., 2023  SURPASS-6 [15] | Low | Low | Low | Low | Low | Low | Objective endpoint; prespecified/complete reporting with low missing-data concern. |
| Giugliano et al., 2021  BEYOND [16] | Low | Some concerns | Low | Low | Low | Some concerns | Open-label; objective weight, but behavioural/titration effects possible. |
| Abreu et al., 2019  SIMPLE [26] | Low | Some concerns | Some concerns | Low | Low | Some concerns | Open-label; objective weight, but behavioural/titration effects possible; missing-data/attrition concern. |
| Aso et al., 2021  IDEAL [27] | Some concerns | Some concerns | Low | Low | Low | Some concerns | Open-label; objective weight, but behavioural/titration effects possible; allocation/randomisation detail unclear. |
| Diamant et al., 2014  4B Study [28] | Low | Some concerns | Low | Low | Low | Some concerns | Open-label; objective weight, but behavioural/titration effects possible. |
| Galindo et al., 2023  DUAL HIGH [29] | Low | Some concerns | Some concerns | Low | Low | Some concerns | Open-label; objective weight, but behavioural/titration effects possible; missing-data/attrition concern. |
| Probstfield et al., 2016  FLAT-SUGAR [30] | Low | Some concerns | Low | Low | Low | Some concerns | Open-label; objective weight, but behavioural/titration effects possible. |
| Mathieu et al., 2014  BEGIN VICTOZA ADD-ON [31] | Low | Some concerns | Low | Low | Low | Some concerns | Open-label; objective weight, but behavioural/titration effects possible. |
| Miya et al., 2018 [32] | Low | Some concerns | Some concerns | Low | Low | Some concerns | Open-label; objective weight, but behavioural/titration effects possible; missing-data/attrition concern. |
| Rodriguez et al., 2024  TRANSITION-T2D [33] | Low | Some concerns | Low | Low | Low | Some concerns | Open-label; objective weight, but behavioural/titration effects possible. |
| Rosenstock et al., 2021  SoliMix [34] | Low | Low | Low | Low | Low | Low | Objective endpoint; prespecified/complete reporting with low missing-data concern. |
| Rosenstock et al., 2014  Harmony 6 [35] | Low | Some concerns | Low | Low | Low | Some concerns | Open-label; objective weight, but behavioural/titration effects possible. |
| Rosenstock et al., 2016  GetGoal Duo-2 [36] | Low | Some concerns | Low | Low | Low | Some concerns | Open-label; objective weight, but behavioural/titration effects possible. |
| Rosenstock et al., 2020 [37] | Low | Some concerns | Low | Low | Low | Some concerns | Open-label; objective weight, but behavioural/titration effects possible. |
| Shao et al., 2014 [38] | Some concerns | Some concerns | Low | Low | Some concerns | Some concerns | Open-label; objective weight, but behavioural/titration effects possible; allocation/randomisation detail unclear; protocol/registry or reporting detail limited; small trial. |
| Yamamoto et al., 2018 [39] | Low | Some concerns | Low | Low | Low | Some concerns | Open-label; objective weight, but behavioural/titration effects possible; small trial. |

*Panel note: Objective outcomes: D4 was judged low unless problematic measurement procedures were identified; open-label designs mainly informed D2 and possible treatment/titration deviations.*

**Table S3C. Outcome-specific RoB 2 assessment for trial-defined hypoglycaemia incidence**

| **Study** | **D1** | **D2** | **D3** | **D4** | **D5** | **Overall** | **Concise rationale** |
| --- | --- | --- | --- | --- | --- | --- | --- |
| Billings et al., 2025  COMBINE 3 [13] | Low | Low | Low | Some concerns | Low | Some concerns | Open-label smbg/symptom-based ascertainment may affect reporting. |
| Billings et al., 2018  DUAL VII [14] | Low | Some concerns | Low | Some concerns | Low | Some concerns | Open-label smbg/symptom-based ascertainment may affect reporting; titration differences possible. |
| Rosenstock et al., 2023  SURPASS-6 [15] | Low | Low | Low | Some concerns | Low | Some concerns | Open-label smbg/symptom-based ascertainment may affect reporting. |
| Giugliano et al., 2021  BEYOND [16] | Low | Some concerns | Low | Some concerns | Low | Some concerns | Open-label smbg/symptom-based ascertainment may affect reporting; titration differences possible. |
| Abreu et al., 2019  SIMPLE [26] | Low | Some concerns | Some concerns | Some concerns | Low | Some concerns | Open-label smbg/symptom-based ascertainment may affect reporting; titration differences possible; missing-data/attrition concern. |
| Aso et al., 2021  IDEAL [27] | Some concerns | Some concerns | Not assessable | Not assessable | Some concerns | Not assessable | No extractable participant-incidence hypoglycaemia result. |
| Diamant et al., 2014  4B Study [28] | Low | Some concerns | Low | Some concerns | Low | Some concerns | Open-label smbg/symptom-based ascertainment may affect reporting; titration differences possible. |
| Galindo et al., 2023  DUAL HIGH [29] | Low | Some concerns | Low | Some concerns | Low | Some concerns | Open-label smbg/symptom-based ascertainment may affect reporting; titration differences possible. |
| Probstfield et al., 2016  FLAT-SUGAR [30] | Low | Some concerns | Low | Low | Low | Some concerns | Open-label treatment/titration may influence events. |
| Mathieu et al., 2014  BEGIN VICTOZA ADD-ON [31] | Low | Some concerns | Not assessable | Not assessable | Some concerns | Not assessable | No extractable participant-incidence hypoglycaemia result. |
| Miya et al., 2018 [32] | Low | Some concerns | Some concerns | Some concerns | Low | Some concerns | Open-label smbg/symptom-based ascertainment may affect reporting; titration differences possible; missing-data/attrition concern. |
| Rodriguez et al., 2024  TRANSITION-T2D [33] | Low | Some concerns | Not assessable | Not assessable | Some concerns | Not assessable | No extractable participant-incidence hypoglycaemia result. |
| Rosenstock et al., 2021  SoliMix [34] | Low | Low | Low | Some concerns | Low | Some concerns | Open-label smbg/symptom-based ascertainment may affect reporting. |
| Rosenstock et al., 2014  Harmony 6 [35] | Low | Some concerns | Low | Some concerns | Low | Some concerns | Open-label smbg/symptom-based ascertainment may affect reporting; titration differences possible. |
| Rosenstock et al., 2016  GetGoal Duo-2 [36] | Low | Some concerns | Low | Some concerns | Low | Some concerns | Open-label smbg/symptom-based ascertainment may affect reporting; titration differences possible. |
| Rosenstock et al., 2020 [37] | Low | Some concerns | Low | Some concerns | Low | Some concerns | Open-label smbg/symptom-based ascertainment may affect reporting; titration differences possible. |
| Shao et al., 2014 [38] | Some concerns | Some concerns | Low | Some concerns | Some concerns | Some concerns | Open-label smbg/symptom-based ascertainment may affect reporting; titration differences possible; allocation/randomisation detail unclear; protocol/registry or reporting detail limited. |
| Yamamoto et al., 2018 [39] | Low | Some concerns | Not assessable | Not assessable | Some concerns | Not assessable | No extractable participant-incidence hypoglycaemia result. |

*Panel note: For assessable trials, participant-incidence hypoglycaemia often depended on SMBG/SMPG, symptoms, diaries, or investigator classification; D4 was generally “some concerns” where open-label monitoring/reporting could influence ascertainment*

**Table S3D. Outcome-specific RoB 2 assessment for severe hypoglycaemia**

| **Study** | **D1** | **D2** | **D3** | **D4** | **D5** | **Overall** | **Concise rationale** |
| --- | --- | --- | --- | --- | --- | --- | --- |
| Billings et al., 2025  COMBINE 3 [13] | Low | Low | Low | Low | Low | Low | Clear severe-event definition and participant-level safety reporting. |
| Billings et al., 2018  DUAL VII [14] | Low | Some concerns | Low | Low | Low | Some concerns | Open-label titration may influence event occurrence. |
| Rosenstock et al., 2023  SURPASS-6 [15] | Low | Low | Low | Low | Low | Low | Clear severe-event definition and participant-level safety reporting. |
| Giugliano et al., 2021  BEYOND [16] | Low | Some concerns | Not assessable | Not assessable | Some concerns | Not assessable | No extractable participant-incidence severe-hypoglycaemia result. |
| Abreu et al., 2019  SIMPLE [26] | Low | Some concerns | Some concerns | Low | Low | Some concerns | Open-label titration may influence event occurrence; missing-data/attrition concern. |
| Aso et al., 2021  IDEAL [27] | Some concerns | Some concerns | Not assessable | Not assessable | Some concerns | Not assessable | No extractable participant-incidence severe-hypoglycaemia result. |
| Diamant et al., 2014  4B Study [28] | Low | Some concerns | Low | Low | Low | Some concerns | Open-label titration may influence event occurrence. |
| Galindo et al., 2023  DUAL HIGH [29] | Low | Some concerns | Low | Low | Low | Some concerns | Open-label titration may influence event occurrence. |
| Probstfield et al., 2016  FLAT-SUGAR [30] | Low | Some concerns | Low | Low | Low | Some concerns | Open-label titration may influence event occurrence. |
| Mathieu et al., 2014  BEGIN VICTOZA ADD-ON [31] | Low | Some concerns | Low | Low | Low | Some concerns | Open-label titration may influence event occurrence. |
| Miya et al., 2018 [32] | Low | Some concerns | Some concerns | Low | Low | Some concerns | Open-label titration may influence event occurrence; missing-data/attrition concern. |
| Rodriguez et al., 2024  TRANSITION-T2D [33] | Low | Some concerns | Low | Low | Low | Some concerns | Open-label titration may influence event occurrence. |
| Rosenstock et al., 2021  SoliMix [34] | Low | Low | Not assessable | Not assessable | Some concerns | Not assessable | No extractable participant-incidence severe-hypoglycaemia result. |
| Rosenstock et al., 2014  Harmony 6 [35] | Low | Some concerns | Low | Low | Low | Some concerns | Open-label titration may influence event occurrence. |
| Rosenstock et al., 2016  GetGoal Duo-2 [36] | Low | Some concerns | Low | Low | Low | Some concerns | Open-label titration may influence event occurrence. |
| Rosenstock et al., 2020 [37] | Low | Some concerns | Low | Low | Low | Some concerns | Open-label titration may influence event occurrence. |
| Shao et al., 2014 [38] | Some concerns | Some concerns | Not assessable | Not assessable | Some concerns | Not assessable | No extractable participant-incidence severe-hypoglycaemia result. |
| Yamamoto et al., 2018 [39] | Low | Some concerns | Low | Low | Low | Some concerns | Open-label titration may influence event occurrence. |

*Panel note: Severe hypoglycaemia was treated as relatively objective when defined by external assistance, cognitive impairment, or medical assistance; D4 was low when a clear participant-level result was reported.*

**Table S3E. Outcome-specific RoB 2 assessment for gastrointestinal adverse events**

| **Study** | **D1** | **D2** | **D3** | **D4** | **D5** | **Overall** | **Concise rationale** |
| --- | --- | --- | --- | --- | --- | --- | --- |
| Billings et al., 2025  COMBINE 3 [13] | Low | Low | Low | Some concerns | Low | Some concerns | Subjective gi symptoms; open-label expectation/reporting bias possible. |
| Billings et al., 2018  DUAL VII [14] | Low | Some concerns | Low | Some concerns | Some concerns | Some concerns | Subjective gi symptoms; open-label expectation/reporting bias possible; incomplete symptom-specific/protocol reporting. |
| Rosenstock et al., 2023  SURPASS-6 [15] | Low | Low | Low | Some concerns | Low | Some concerns | Subjective gi symptoms; open-label expectation/reporting bias possible. |
| Giugliano et al., 2021  BEYOND [16] | Low | Some concerns | Not assessable | Not assessable | Some concerns | Not assessable | No usable participant-level GI adverse-event incidence. |
| Abreu et al., 2019  SIMPLE [26] | Low | Some concerns | Some concerns | Some concerns | Low | Some concerns | Subjective gi symptoms; open-label expectation/reporting bias possible; missing-data/attrition concern. |
| Aso et al., 2021  IDEAL [27] | Some concerns | Some concerns | Not assessable | Not assessable | Some concerns | Not assessable | No usable participant-level GI adverse-event incidence. |
| Diamant et al., 2014  4B Study [28] | Low | Some concerns | Low | Some concerns | Some concerns | Some concerns | Subjective gi symptoms; open-label expectation/reporting bias possible; incomplete symptom-specific/protocol reporting. |
| Galindo et al., 2023  DUAL HIGH [29] | Low | Some concerns | Low | Some concerns | Low | Some concerns | Subjective gi symptoms; open-label expectation/reporting bias possible. |
| Probstfield et al., 2016  FLAT-SUGAR [30] | Low | Some concerns | Low | Some concerns | Some concerns | Some concerns | Subjective gi symptoms; open-label expectation/reporting bias possible; incomplete symptom-specific/protocol reporting. |
| Mathieu et al., 2014  BEGIN VICTOZA ADD-ON [31] | Low | Some concerns | Low | Some concerns | Some concerns | Some concerns | Subjective gi symptoms; open-label expectation/reporting bias possible; incomplete symptom-specific/protocol reporting. |
| Miya et al., 2018 [32] | Low | Some concerns | Some concerns | Some concerns | Low | Some concerns | Subjective gi symptoms; open-label expectation/reporting bias possible; missing-data/attrition concern. |
| Rodriguez et al., 2024  TRANSITION-T2D [33] | Low | Some concerns | Not assessable | Not assessable | Some concerns | Not assessable | No usable participant-level GI adverse-event incidence. |
| Rosenstock et al., 2021  SoliMix [34] | Low | Low | Low | Some concerns | Low | Some concerns | Subjective gi symptoms; open-label expectation/reporting bias possible. |
| Rosenstock et al., 2014  Harmony 6 [35] | Low | Some concerns | Low | Some concerns | Low | Some concerns | Subjective gi symptoms; open-label expectation/reporting bias possible. |
| Rosenstock et al., 2016  GetGoal Duo-2 [36] | Low | Some concerns | Low | Some concerns | Low | Some concerns | Subjective gi symptoms; open-label expectation/reporting bias possible. |
| Rosenstock et al., 2020 [37] | Low | Some concerns | Low | Some concerns | Some concerns | Some concerns | Subjective gi symptoms; open-label expectation/reporting bias possible; incomplete symptom-specific/protocol reporting. |
| Shao et al., 2014 [38] | Some concerns | Some concerns | Low | Some concerns | Some concerns | Some concerns | Subjective gi symptoms; open-label expectation/reporting bias possible; incomplete symptom-specific/protocol reporting; allocation/randomisation detail unclear. |
| Yamamoto et al., 2018 [39] | Low | Some concerns | Not assessable | Not assessable | Some concerns | Not assessable | No usable participant-level GI adverse-event incidence. |

*Panel note: GI adverse events were treated as subjective safety outcomes; D4 was generally “some concerns” in open-label trials unless no usable GI AE result was available.*

**Table S3F. Outcome-specific RoB 2 assessment for withdrawals due to adverse events**

| **Study** | **D1** | **D2** | **D3** | **D4** | **D5** | **Overall** | **Concise rationale** |
| --- | --- | --- | --- | --- | --- | --- | --- |
| Billings et al., 2025  COMBINE 3 [13] | Low | Low | Low | Some concerns | Low | Some concerns | Open-label ae attribution/discontinuation potentially expectation-sensitive. |
| Billings et al., 2018  DUAL VII [14] | Low | Some concerns | Low | Some concerns | Low | Some concerns | Open-label ae attribution/discontinuation potentially expectation-sensitive. |
| Rosenstock et al., 2023  SURPASS-6 [15] | Low | Low | Low | Some concerns | Low | Some concerns | Open-label ae attribution/discontinuation potentially expectation-sensitive. |
| Giugliano et al., 2021  BEYOND [16] | Low | Some concerns | Low | Some concerns | Low | Some concerns | Open-label ae attribution/discontinuation potentially expectation-sensitive. |
| Abreu et al., 2019  SIMPLE [26] | Low | Some concerns | Low | Some concerns | Low | Some concerns | Open-label ae attribution/discontinuation potentially expectation-sensitive. |
| Aso et al., 2021  IDEAL [27] | Some concerns | Some concerns | Low | Some concerns | Low | Some concerns | Open-label ae attribution/discontinuation potentially expectation-sensitive; allocation/randomisation detail unclear. |
| Diamant et al., 2014  4B Study [28] | Low | Some concerns | Low | Some concerns | Low | Some concerns | Open-label ae attribution/discontinuation potentially expectation-sensitive. |
| Galindo et al., 2023  DUAL HIGH [29] | Low | Some concerns | Low | Some concerns | Low | Some concerns | Open-label ae attribution/discontinuation potentially expectation-sensitive. |
| Probstfield et al., 2016  FLAT-SUGAR [30] | Low | Some concerns | Low | Some concerns | Low | Some concerns | Open-label ae attribution/discontinuation potentially expectation-sensitive. |
| Mathieu et al., 2014  BEGIN VICTOZA ADD-ON [31] | Low | Some concerns | Low | Some concerns | Low | Some concerns | Open-label ae attribution/discontinuation potentially expectation-sensitive. |
| Miya et al., 2018 [32] | Low | Some concerns | Low | Some concerns | Low | Some concerns | Open-label ae attribution/discontinuation potentially expectation-sensitive. |
| Rodriguez et al., 2024  TRANSITION-T2D [33] | Low | Some concerns | Low | Some concerns | Low | Some concerns | Open-label ae attribution/discontinuation potentially expectation-sensitive. |
| Rosenstock et al., 2021  SoliMix [34] | Low | Low | Low | Some concerns | Low | Some concerns | Open-label ae attribution/discontinuation potentially expectation-sensitive. |
| Rosenstock et al., 2014  Harmony 6 [35] | Low | Some concerns | Low | Some concerns | Low | Some concerns | Open-label ae attribution/discontinuation potentially expectation-sensitive. |
| Rosenstock et al., 2016  GetGoal Duo-2 [36] | Low | Some concerns | Low | Some concerns | Low | Some concerns | Open-label ae attribution/discontinuation potentially expectation-sensitive. |
| Rosenstock et al., 2020 [37] | Low | Some concerns | Low | Some concerns | Low | Some concerns | Open-label ae attribution/discontinuation potentially expectation-sensitive. |
| Shao et al., 2014 [38] | Some concerns | Some concerns | Low | Some concerns | Some concerns | Some concerns | Open-label ae attribution/discontinuation potentially expectation-sensitive; allocation/randomisation detail unclear; protocol/registry or reporting detail limited. |
| Yamamoto et al., 2018 [39] | Low | Some concerns | Low | Some concerns | Low | Some concerns | Open-label ae attribution/discontinuation potentially expectation-sensitive. |

*Panel note: Withdrawals due to adverse events were objectively counted, but attribution and discontinuation decisions can be influenced by open-label treatment assignment and known gastrointestinal tolerability*, ae = adverse event.

**Fig. S1. Summary risk-of-bias plot for change in HbA1c (%).** Distribution of Cochrane RoB 2 judgements across the five bias domains and overall risk of bias for the included randomized controlled trials. Green indicates low risk of bias and yellow indicates some concerns.

**Fig. S2. Traffic-light plot of study-level risk-of-bias judgements for change in HbA1c (%).** Domain-level and overall Cochrane RoB 2 judgements for each included randomized controlled trial. D1, bias arising from the randomization process; D2, bias due to deviations from intended interventions; D3, bias due to missing outcome data; D4, bias in measurement of the outcome; D5, bias in selection of the reported result. Green indicates low risk of bias and yellow indicates some concerns [13-16,26-39].

**Fig. S3. Change in HbA1c (%) with incretin-based injectable regimens versus intensified insulin therapy, stratified by clinical pathway.** Forest plot of mean differences (MDs) in HbA1c change from baseline to end of follow-up, stratified by intensification trials and simplification trials. Pooled effects were calculated using a random-effects generic inverse-variance model. Negative MD values favour incretin-based injectable regimens. Squares represent study-specific effect estimates and horizontal lines indicate 95% confidence intervals (CIs); diamonds represent subgroup and overall pooled estimates [13-16,26-37,39].

**Fig. S4. Sensitivity analysis for change in HbA1c (%) excluding IDEAL.** Forest plot of mean differences (MDs) in HbA1c change from baseline to end of follow-up after excluding IDEAL, for which the variance estimate was derived from the reported p value. Pooled effects were calculated using a random-effects generic inverse-variance model. Negative MD values favour incretin-based injectable regimens. Squares represent study-specific effect estimates and horizontal lines indicate 95% confidence intervals (CIs); the diamond represents the pooled effect estimate and its 95% CI [13-16,26,28-37,39].

**Fig. S5. Sensitivity analysis for change in HbA1c (%) excluding SURPASS-6.** Forest plot of mean differences (MDs) in HbA1c change from baseline to end of follow-up after excluding SURPASS-6, the pharmacologically distinct tirzepatide trial. Pooled effects were calculated using a random-effects generic inverse-variance model. Negative MD values favour incretin-based injectable regimens. Squares represent study-specific effect estimates and horizontal lines indicate 95% confidence intervals (CIs); the diamond represents the pooled effect estimate and its 95% CI [13,14,16,26-37,39].

**Fig. S6. Sensitivity analysis for change in HbA1c (%) excluding trials with CI/ETD-derived standard-error inputs.** Forest plot of mean differences (MDs) in HbA1c change from baseline to end of follow-up after excluding trials in which standard errors were derived from reported confidence intervals or estimated treatment differences. Pooled effects were calculated using a random-effects generic inverse-variance model. Negative MD values favour incretin-based injectable regimens. Squares represent study-specific effect estimates and horizontal lines indicate 95% confidence intervals (CIs); the diamond represents the pooled effect estimate and its 95% CI [13,16,26-30,32-37,39].

**Fig. S7. Sensitivity analysis for change in HbA1c (%) excluding small or highly imprecise trials.** Forest plot of mean differences (MDs) in HbA1c change from baseline to end of follow-up after excluding small or highly imprecise trials. Pooled effects were calculated using a random-effects generic inverse-variance model. Negative MD values favour incretin-based injectable regimens. Squares represent study-specific effect estimates and horizontal lines indicate 95% confidence intervals (CIs); the diamond represents the pooled effect estimate and its 95% CI [13-16,27-31,33-37].

**Fig. S8. Sensitivity analysis for trial-defined hypoglycaemia incidence excluding very small trials.** Forest plot of risk ratios (RRs) for the proportion of participants experiencing at least one trial-defined hypoglycaemic event after excluding very small trials. Pooled effects were calculated using a random-effects Mantel-Haenszel model. RR values <1 favour incretin-based injectable regimens. Squares represent study-specific effect estimates and horizontal lines indicate 95% confidence intervals (CIs); the diamond represents the pooled effect estimate and its 95% CI [13-16,26,28-30,34-37].

**Fig. S9. Sensitivity analysis for trial-defined hypoglycaemia incidence excluding FLAT-SUGAR.** Forest plot of risk ratios (RRs) for the proportion of participants experiencing at least one trial-defined hypoglycaemic event after excluding FLAT-SUGAR, which used continuous-glucose-monitoring-based ascertainment. Pooled effects were calculated using a random-effects Mantel-Haenszel model. RR values <1 favour incretin-based injectable regimens. Squares represent study-specific effect estimates and horizontal lines indicate 95% confidence intervals (CIs); the diamond represents the pooled effect estimate and its 95% CI [13-16,26,28,29,32,34-38].

**Table S4: Change in body weight: study-level extracted data**

| **Study** | **Incretin Arm Mean Change** | **Incretin SD** | **Incretin N** | **Comparator Arm Mean Change** | **Comparator SD** | **Comparator N** | **Timepoint** | **Notes on SD Extraction** |
| --- | --- | --- | --- | --- | --- | --- | --- | --- |
| Billings et al., 2025  COMBINE 3 [13] | -3.56 | 5.35 | 340 | 3.16 | 5.89 | 328 | Week 52 | SD calculated from SE of change (0.29 & 0.32). |
| Billings et al., 2018  DUAL VII [14] | -0.9 | 3.7* | 252 | 2.6 | 3.7* | 254 | Week 26 | SD imputed from ETD 95% CI (width 1.3 -> SE~0.33) assuming equal variance. |
| Rosenstock et al., 2023  SURPASS-6 [15] | -9 | 7.5 | 716 | 3.2 | 14.3 | 708 | Week 52 | SD calculated from 95% CIs of the change. |
| Giugliano et al., 2021  BEYOND [16] | -1.9 | 4.3 | 102 | 0.3 | 1.5 | 101 | Week 26 | Mean (SD) change explicitly reported. |
| Abreu et al., 2019  SIMPLE [26] | -0.6 | 5.6 | 54 | 3.1 | 5.5 | 56 | Week 26 | SD calculated from 95% CIs of the change. |
| Aso et al., 2021  IDEAL [27] | -2.79 | NR | 24 | -0.78 | NR | 28 | Week 52 | SD of change **NR**. Baseline SDs provided here* for imputation (r required). |
| Diamant et al., 2014  4B Study [28] | -2.5 | 4.7 | 247 | 2.1 | 3.2 | 263 | Week 30 | SD calculated from SE of change (0.3 & 0.2). |
| Galindo et al., 2023  DUAL HIGH [29] | 1.24 | 8.33 | 72 | 5.84 | 6.18 | 73 | Week 26 | Mean (SD) change explicitly reported. |
| Probstfield et al., 2016  FLAT-SUGAR [30] | -4.8 | 3.3 | 45 | 0.7 | 3.3 | 44 | Week 26 | Mean (SD) change explicitly reported. |
| Mathieu et al., 2014  BEGIN VICTOZA ADD-ON [31] | -2.8 | 3.8 | 88 | 0.9 | 2.5 | 89 | Week 26 | Mean (SD) change explicitly reported. |
| Miya et al., 2018 [32] | -2.5 | 1.8 | 11 | 0.6 | 1.8 | 15 | Week 12 | Mean (SD) change explicitly reported. |
| Rodriguez et al., 2024  TRANSITION-T2D [33] | -8.9 | 3.4 | 40 | 1.5 | 3.7 | 20 | Week 26 | SD calculated from 95% CIs of the change. |
| Rosenstock et al., 2021  SoliMix [34] | -0.7 | 4.2 | 443 | 1.2 | 4.2 | 444 | Week 26 | SD calculated from SE of change (0.2 & 0.2). |
| Rosenstock et al., 2014  Harmony 6 [35] | -0.73 | 3.2 | 285 | 0.81 | 3.2 | 281 | Week 26 | SD calculated from SE of change (0.19 & 0.19). |
| Rosenstock et al., 2016  GetGoal Duo-2 [36] | -0.6 | 5.2 | 295 | 1.4 | 5.2 | 295 | Week 26 | SD calculated from SE of change (0.3 & 0.3). Comparator is Thrice-Daily arm. |
| Rosenstock et al., 2020 [37] | -2 | 3.7 | 349 | 2.4 | 3.8 | 352 | Week 26 | SD calculated from SE of change (0.2 & 0.2). |
| Shao et al., 2014 [38] | -7.77 | 3.4 | 30 | 3.27 | 2.7* | 30 | Week 12 | **Mean Change calculated from Pre/Post Means. |
| Yamamoto et al., 2018 [39] | -2.5 | 3.6 | 12 | 2.8 | 2.8 | 13 | Week 24 | Mean (SD) change explicitly reported. |

*** Indicates standard deviation (SD) values that were not explicitly reported in the original trial and had to be imputed or derived.**

**Fig. S10. HbA1c target achievement with incretin-based injectable regimens versus intensified insulin therapy.** Forest plot of risk ratios (RRs) for the proportion of participants achieving the trial-defined HbA1c target at end of follow-up, comparing incretin-based injectable regimens with intensified insulin strategies. Pooled effects were calculated using a random-effects Mantel-Haenszel model. RR values >1 favour incretin-based injectable regimens. Squares represent study-specific effect estimates and horizontal lines indicate 95% confidence intervals (CIs); the diamond represents the pooled effect estimate and its 95% CI [13-16,26-28,31,34-37].

**Table S5. HbA1c target achievement at endpoint: study-level extracted data**

| Study | Incretin Arm (n / N) | Comparator Arm (n / N) | Target | Notes |
| --- | --- | --- | --- | --- |
| Billings et al., 2025  COMBINE 3 [13] | 227 / 340 † | 190 / 339 † | <7.0% | *n* derived from estimated % (66.9% vs 56.0%). |
| Billings et al., 2018  DUAL VII [14] | 166 / 252 † | 170 / 254 † | <7.0% | *n* derived from reported % (66% vs 67%). |
| Rosenstock et al., 2023  SURPASS-6 [15] | 483 / 716 | 256 / 708 | <7.0% | Explicit *n*/*N* reported. Pooled Tirzepatide vs Lispro. |
| Giugliano et al., 2021  BEYOND [16] | 19 / 102 † | 19 / 101 † | ≤7.0% | *n* derived from reported % (19% vs 19%). Fixed-combo vs BBI. |
| Abreu et al., 2019  SIMPLE [26] | 24 / 54 † | 11 / 56 † | ≤7.0% | *n* derived from reported % (44% vs 20%). mITT population. |
| Aso et al., 2021  IDEAL [27] | 10 / 24 † | 5 / 28 † | <7.0% | *n* derived from reported % (40% vs 19%). |
| Diamant et al., 2014  4B Study [28] | 123 / 247 † | 129 / 263 † | ≤7.0% | *n* derived from % (49.6% vs 49.0%). PP population used for secondary outcomes in this trial. |
| Mathieu et al., 2014  BEGIN VICTOZA ADD-ON [31] | 51 / 88 | 40 / 89 | <7.0% | Explicit *n*/*N* reported in text. |
| Rosenstock et al., 2021  SoliMix [34] | 122 / 443 | 55 / 444 | <7.0% | Explicit *n*/*N* reported. (27.5% vs 12.4%). |
| Rosenstock et al., 2014  Harmony 6 [35] | 86 / 285 † | 70 / 281 † | <7.0% | *n* derived from reported % (30% vs 25%). |
| Rosenstock et al., 2016  GetGoal Duo-2 [36] | 123 / 292 | 145 / 295 | <7.0% | Explicit *n*/*N* reported. Comparator is Glulisine TID (Basal-Bolus). |
| Rosenstock et al., 2020 [37] | 244 / 402 | 255 / 412 | <7.0% | Explicit *n*/*N* reported. |

• Target Definition: Studies used either <7.0% or ≤7.0% as the target. This is specified in the "Target" column.

• Derived Counts: Where a study reported only the percentage (%) and total N, the number of participants (n) was calculated (n=%×N) and rounded to the nearest whole number. These are marked with a dagger (†).

• Populations: Data primarily reflects the Full Analysis Set (FAS) or Intent-to-Treat (ITT) population unless the study specified per-protocol (PP) for this secondary endpoint (noted for the 4B Study).

**Table S6. Total daily insulin dose: study-level narrative synthesis**

| **Study** | **Comparator Strategy** | **Timepoint** | **Measure Type** | **Incretin Arm Dose (Value ± Dispersion; N)** | **Comparator Arm Dose (Value ± Dispersion; N)** | **Direction** | **Notes** |
| --- | --- | --- | --- | --- | --- | --- | --- |
| Billings et al., 2025  COMBINE 3 [13] | Basal-Bolus | Week 52 | Endpoint (Mean ± SE) | 196 ± 12; n=340 | 466 ± 12; n=328 | Favours Incretin | Data reported as Units/Week, not Units/Day; Endpoint data. |
| Billings et al., 2018  DUAL VII [14] | Basal-Bolus | Week 26 | Endpoint (Mean) | 40; n=252 | 84; n=254 | Favours Incretin | Endpoint data; SD not reported in text. |
| Rosenstock et al., 2023  SURPASS-6 [15] | Basal-Bolus | Week 52 | Endpoint (Geometric Mean) | 13 IU (Basal only); n=717 | 112 IU (Total); n=708 | Favours Incretin | Reports geometric means; comparator dose includes high volume of prandial insulin. |
| Giugliano et al., 2021  BEYOND [16] | Basal-Bolus | Week 26 | Change from Baseline (Mean ± SD) | -27.1 ± 14.2; n=102 | +12.3 ± 7.4; n=101 | Favours Incretin | Reports Change scores; cannot pool with endpoint studies without correlation assumptions. |
| Abreu et al., 2019  SIMPLE [26] | Basal-Bolus | Week 26 | Endpoint (Geometric Mean, 95% CI) | 59.4 (49.7–70.9); n=54 | 94.5 (79.2–112.7); n=56 | Favours Incretin | Reports geometric means rather than arithmetic means; Endpoint data. |
| Aso et al., 2021  IDEAL [27] | Premix | Week 52 | Endpoint (Mean ± SD) | 15.1 ± 8.1; n=24 | 20.5 ± 9.7; n=28 | Favours Incretin | Endpoint data; Comparator is Premix. |
| Diamant et al., 2014  4B Study [28] | Basal-Bolus | Week 30 | Endpoint (Mean ± SD) | 56.9 ± 29.4; n=247 | 93.7 ± 53.9; n=263 | Favours Incretin | Endpoint data. |
| Galindo et al., 2023  DUAL HIGH [29] | Basal-Bolus | Week 26 | NR | NR | NR | Favours Incretin | Text states "lower insulin requirements" with Incretin, but specific final total daily dose values are not reported. |
| Probstfield et al., 2016  FLAT-SUGAR [30] | Basal-Bolus | Week 26 | Endpoint (Mean) | ~58; n=49 | ~88; n=47 | Favours Incretin | Endpoint data; values approximated from text/table components. |
| Mathieu et al., 2014  BEGIN VICTOZA ADD-ON [31] | Basal-Plus | Week 26 | Endpoint (Mean U/kg) | 0.65 U/kg; n=88 | 0.85 U/kg; n=89 | Favours Incretin | Data reported as Units/kg, not Units/day. |
| Miya et al., 2018 [32] | Basal-Bolus | Week 12 | Change from Baseline (Mean ± SD) | -12.7 ± 3.4; n=11 | +0.7 ± 4.8; n=15 | Favours Incretin | Reports Change scores; very small sample size. |
| Rodriguez et al., 2024  TRANSITION-T2D [33] | Basal-Bolus | Week 26 | Change from Baseline (Mean, 95% CI) | -37.3 (-41.1 to -33.6); n=40 | +5.2 (-0.3 to 10.6); n=20 | Favours Incretin | Reports Change scores; SD must be imputed from CI. |
| Rosenstock et al., 2021  SoliMix [34] | Premix | Week 26 | Change from Baseline (Mean ± SE) | -0.7 ± 0.2; n=443 | +1.2 ± 0.2; n=444 | Favours Incretin | Reports Change scores; Comparator is Premix (not Basal-Bolus). |
| Rosenstock et al., 2014  Harmony 6 [35] | Basal-Bolus | Week 26 | Endpoint (Mean) | 53.2; n=285 | 81.2; n=281 | Favours Incretin | Endpoint data; SD not explicitly reported in text for total dose (components reported separately). |
| Rosenstock et al., 2016  GetGoal Duo-2 [36] | Basal-Bolus (TID) | Week 26 | Endpoint (Mean ± SD) | 67 ± 32; n=298 | 81 ± 34; n=294 | Favours Incretin | Endpoint data; Comparator is Glulisine TID arm. |
| Rosenstock et al., 2020 [37] | Basal-Bolus | Week 26 | Endpoint (Mean ± SD) | 69.0 ± 33.2; n=342 | 130.4 ± 61.1; n=341 | Favours Incretin | Endpoint data; very large disparity in final doses. |
| Shao et al., 2014 [38] | Basal-Bolus | Week 12 | NR | NR | NR | NR | Final total daily insulin dose data not reported. |
| Yamamoto et al., 2018 [39] | Basal-Bolus | Week 24 | Endpoint (Mean ± SD) | 10.4 ± 8.4; n=12 | 25.6 ± 11.1; n=13 | Favours Incretin | Endpoint data; very small sample size. |

**Values are presented as reported in the source trial and were not pooled because studies reported insulin dose using heterogeneous units, timepoints, and summary statistics. Direction of effect was judged descriptively.**

**Fig. S11. Severe hypoglycaemia with incretin-based injectable regimens versus intensified insulin therapy.** Forest plot of risk ratios (RRs) for the proportion of participants experiencing at least one severe hypoglycaemic event, defined as Level 3 hypoglycaemia or hypoglycaemia requiring assistance. Pooled effects were calculated using a fixed-effect Mantel-Haenszel model. RR values <1 favour incretin-based injectable regimens. Squares represent study-specific effect estimates and horizontal lines indicate 95% confidence intervals (CIs); the diamond represents the pooled effect estimate and its 95% CI [13-15,26,28,35,37].

**Fig. S12. Sensitivity analysis for severe hypoglycaemia using risk difference.** Forest plot of risk differences (RDs) for the proportion of participants experiencing at least one severe hypoglycaemic event, defined as Level 3 hypoglycaemia or hypoglycaemia requiring assistance. Pooled effects were calculated using a fixed-effect Mantel-Haenszel model. RD values <0 favour incretin-based injectable regimens. Squares represent study-specific effect estimates and horizontal lines indicate 95% confidence intervals (CIs); the diamond represents the pooled effect estimate and its 95% CI [13-15,26,28,35,37].

**Table S7: Severe hypoglycaemia: study-level extracted data**

| **Study** | **Incretin Arm**  **(n / N)** | **Comparator Arm**  **(n / N)** | **Definition / Notes** |
| --- | --- | --- | --- |
| Billings et al., 2025  COMBINE 3 [13] | 4 / 340 | 4 / 328 | **Level 3**: Severe cognitive impairment requiring external assistance. |
| Billings et al., 2018  DUAL VII [14] | 3 / 252 | 4 / 254 | **Severe**: ADA classification (requiring assistance). (1.2% vs 1.6% of patients). |
| Rosenstock et al., 2023  SURPASS-6 [15] | 3 / 717 | 30 / 708 | **Severe**: Severe cognitive impairment requiring assistance. Pooled Tirzepatide vs Lispro. |
| Giugliano et al., 2021  BEYOND [16] | NR | NR | Text lumps Level 2 and 3. Specific Level 3 count NR. |
| Abreu et al., 2019  SIMPLE [26] | 1/54 | 2/56 | **Severe**: Symptoms needing someone else's help to recover. |
| Aso et al., 2021  IDEAL [27] | NR | NR | Reports rates of "confirmed hypoglycaemia." Does not explicitly report severe/assistance-requiring counts. |
| Diamant et al., 2014  4B Study [28] | 0 / 315 | 2 / 312 | **Major**: Requiring third-party assistance. |
| Galindo et al., 2023  DUAL HIGH [29] | 0 / 72 | 0 / 73 | **Level 3**: Reported as 0.0 (0,0) for both. |
| Probstfield et al., 2016  FLAT-SUGAR [30] | 0 / 52 | 0 / 50 | **Severe**: Hypoglycaemia requiring medical assistance. |
| Mathieu et al., 2014  BEGIN VICTOZA ADD-ON [31] | 0 / 87 | 0 / 86 | **Severe**: Requiring assistance. |
| Miya et al., 2018 [32] | 0 / 16 | 0 / 15 | Text states "Cases of severe hypoglycaemia were not observed in either group." |
| Rodriguez et al., 2024  TRANSITION-T2D [33] | 0 / 40 | NR / 20 | **Event Count Only**: Semaglutide had 0 events. MDI had "two Level 3 events." Participant count not confirmed (could be 1 or 2 patients). |
| Rosenstock et al., 2021  SoliMix [34] | NR / 443 | NR / 444 | **Event Count Only**: 1 severe episode (iGlarLixi) vs 2 severe episodes (BIAsp 30). Participant count not explicitly confirmed. |
| Rosenstock et al., 2014  Harmony 6 [35] | 0 / 285 | 2 / 281 | **Severe**: Requiring assistance. (0% vs 0.7%). |
| Rosenstock et al., 2016  GetGoal Duo-2 [36] | 0 / 298 | 0 / 294 | **Severe**: Requiring assistance. Comparator is Basal-Bolus (TID). (Basal-Plus arm had 2 events). |
| Rosenstock et al., 2020 [37] | 9 / 400 | 22 / 413 | **Severe**: Requiring third-party intervention. |
| Shao et al., 2014 [38] | NR | NR | Reports "symptomatic" hypoglycaemia (0 vs 3 patients) but does not distinguish "severe." |
| Yamamoto et al., 2018 [39] | 0 / 12 | 0 / 13 | Text states "Cases of severe hypoglycaemia were not observed in either group." |

**Abbreviations: NR:** Not Reported (or data not extractable as participant incidence); ADA: American Diabetes Association.

**Table S8: Gastrointestinal adverse events: study-level extracted data and reporting notes**

| **Study** | **Comparator Strategy** | **Nausea (Incretin vs Comp)** | **Vomiting (Incretin vs Comp)** | **Diarrhea (Incretin vs Comp)** | **Notes (Definition, Window, etc.)** |
| --- | --- | --- | --- | --- | --- |
| Billings et al., 2025  COMBINE 3 [13] | Basal-Bolus | 74 / 340 vs 8 / 328 | 34 / 340 vs 9 / 328 | 43 / 340 vs 18 / 328 | TEAEs ≥5% reported. Safety analysis set. |
| Billings et al., 2018  DUAL VII [14] | Basal-Bolus | 28 / 252† vs 8 / 254† | NR | NR | derived from % (11.1% vs 3.2%). Vomiting/Diarrhoea not listed in common AEs. |
| Rosenstock et al., 2023  SURPASS-6 [15] | Basal-Bolus | 143 / 717 vs 8 / 708 | 62 / 717 vs 4 / 708 | 91 / 717 vs 17 / 708 | Pooled Tirzepatide arm vs Lispro. TEAEs ≥5%. |
| Giugliano et al., 2021  BEYOND [16] | Basal-Bolus | NR | NR | NR | Specific counts NR. Text notes dropouts due to GI events were higher in fixed-combo arm. |
| Abreu et al., 2019  SIMPLE [26] | Basal-Bolus | 32 / 54 vs 16 / 56 | 20 / 54 vs 10 / 56 | 21 / 54 vs 13 / 56 | Events proactively elicited at each visit, leading to higher rates in both arms. |
| Aso et al., 2021  IDEAL [27] | Premix | NR | NR | NR | 3 discontinued for nausea in IDeg+Lira arm. Total incidence NR. |
| Diamant et al., 2014  4B Study [28] | Basal-Bolus | NR | NR | NR | Specific symptom counts NR. Text states overall GI events were 47% (Exenatide) vs 13% (Lispro). |
| Galindo et al., 2023  DUAL HIGH [29] | Basal-Bolus | 27 / 72 vs 3 / 73 | 9 / 72 vs 1 / 73 | 8 / 72 vs 6 / 73 | Cumulative events Weeks 1–26. |
| Probstfield et al., 2016  FLAT-SUGAR [30] | Basal-Bolus | NR | NR | NR | 13 events in GLP-1 arm were GI-related; specific symptom counts NR. |
| Mathieu et al., 2014  BEGIN VICTOZA ADD-ON [31] | Basal-Plus | 18 / 87 vs NR | NR | NR | Data represents "Probably Related" AEs only. Total TEAEs not provided in excerpt. |
| Miya et al., 2018 [32] | Basal-Bolus | 6 / 16 vs 0 / 15 | NR | NR | Reported as "Gastrointestinal symptoms" combined (37.5% vs 0%). |
| Rodriguez et al., 2024  TRANSITION-T2D [33] | Basal-Bolus | NR | NR | NR | Text states "no significant between-group differences" for AEs. |
| Rosenstock et al., 2021  SoliMix [34] | Premix | 34 / 443† vs 0 / 444 | NR | NR | derived from % (7.7% vs 0%). Nausea was the most common AE. |
| Rosenstock et al., 2014  Harmony 6 [35] | Basal-Bolus | 32 / 285 vs 4 / 281 | 19 / 285 vs 4 / 281 | 37 / 285 vs 12 / 281 | TEAEs occurring in ≥5%. |
| Rosenstock et al., 2016  GetGoal Duo-2 [36] | Basal-Bolus (TID) | 75 / 298 vs 3 / 294 | 26 / 298 vs 6 / 294 | 20 / 298 vs 4 / 294 | TEAEs. Comparator is Glulisine TID arm. |
| Rosenstock et al., 2020 [37] | Basal-Bolus | NR | NR | NR | Total GI events: 26% (Albiglutide) vs 13% (Lispro). Specifics NR. |
| Shao et al., 2014 [38] | Basal-Bolus | 6 / 30 vs NR | 2 / 30 vs NR | 1 / 30 vs NR | Comparator symptom-specific GI AE counts not explicitly reported. |
| Yamamoto et al., 2018 [39] | Basal-Bolus | NR | NR | NR | Data not reported. |

**Abbreviations:**

• **NR:** Not Reported (data not available in the provided excerpts).

• **TEAE:** Treatment-Emergent Adverse Event.

• **†:** Count () derived from reported percentage.

**Gastrointestinal Adverse Events (GI AEs).**

Notes on Data Selection:

• Data Type: Data reflects Treatment-Emergent Adverse Events (TEAEs) unless otherwise noted.

• Reporting: Many studies reported GI events as a cluster (e.g., "Gastrointestinal disorders") or only reported the most frequent event (usually nausea). Where vomiting or diarrhea were not explicitly reported in the text or tables, they are marked as NR (Not Reported).

• Comparator: Basal-Bolus (or Premix in SoliMix/IDEAL) arms generally had very low rates of GI events compared to Incretin arms.

• Derivation Where only percentages were reported, event counts were derived as n = percentage × denominator and rounded to the nearest whole number; derived values are marked with †.

**Table S9: Withdrawals due to adverse events: study-level extracted data.**

| **Study** | **Incretin Arm**  **(n / N)** | **Comparator Arm**  **(n / N)** | **Label Used in Source** |
| --- | --- | --- | --- |
| Billings et al., 2025  COMBINE 3 [13] | 16 / 340 | 9 / 328 | Adverse event that led to premature discontinuation |
| Billings et al., 2018  DUAL VII [14] | 7 / 252 | 2 / 254 | Discontinued treatment due to adverse event |
| Rosenstock et al., 2023  SURPASS-6 [15] | 43 / 717 | 17 / 708 | Adverse event leading to study treatment discontinuation |
| Giugliano et al., 2021  BEYOND [16] | 4 / 102 | 0 / 101 | Discontinued for adverse events |
| Abreu et al., 2019  SIMPLE [26] | 1 / 59 | 0 / 61 | Discontinued [drug] because of adverse events |
| Aso et al., 2021  IDEAL [27] | 4 / 24 | 0 / 28 | Discontinued due to [nausea/diarrhoea] vs [None listed for comparator] |
| Diamant et al., 2014  4B Study [28] | 17 / 315 | 8 / 312 | Discontinued [intervention due to] adverse event |
| Galindo et al., 2023  DUAL HIGH [29] | 0 / 72 | 1 / 73 | Discontinued intervention... Adverse events |
| Probstfield et al., 2016  FLAT-SUGAR [30] | 3 / 52 | 1 / 50 | Discontinued due to [Specific AEs listed: death, numbness, side effects vs pancreatic cancer] |
| Mathieu et al., 2014  BEGIN VICTOZA ADD-ON [31] | 5 / 87 | 1 / 86 | Withdrawn due to AEs |
| Miya et al., 2018 [32] | 2 / 16 | 0 / 15 | Discontinued treatment prematurely because of [AEs] |
| Rodriguez et al., 2024  TRANSITION-T2D [33] | 2 / 40 | 1 / 20 | Adverse event leading to drug discontinuation |
| Rosenstock et al., 2021  SoliMix [34] | 3 / 442* | 3 / 441* | AE leading to treatment discontinuation *Derived from reported % (0.7% in both) |
| Rosenstock et al., 2014  Harmony 6 [35] | 15 / 285 | 1 / 281 | Any AE leading to withdrawal |
| Rosenstock et al., 2016  GetGoal Duo-2 [36] | 15 / 298 | 3 / 294 | Treatment-emergent AE leading to permanent discontinuation |
| Rosenstock et al., 2020 [37] | 14 / 402 | 9 / 412 | On-therapy AE leading to discontinuation of study treatment |
| Shao et al., 2014 [38] | 0 / 30 | 0 / 30 | Withdrew from the study due to adverse events |
| Yamamoto et al., 2018 [39] | 2 / 15 | 1 / 16 | Withdrawn [due to Lung cancer/Cerebral infarction vs Cerebral infarction] |

- SoliMix: states discontinuation due to AE was "0.7% iGlarLixi and 0.7% BIAsp 30". With group sizes of 443 and 444, this calculates to approximately 3 participants per arm.
- SURPASS-6: Data represents "Study Treatment Discontinuation" (43 vs 17). The study also reported "Study Discontinuation" due to AEs (12 vs 15), but treatment discontinuation is the standard measure for tolerability in this drug class.
- Comparator: For GetGoal Duo-2, the Basal-Bolus (Glulisine TID) arm data is used (n=294). The Basal-Plus (Glulisine OD) arm had 2 withdrawals (0.7%).

**Note:** Denominators are presented as reported in each source trial and may reflect safety, randomised, ITT, or available-case populations. Because definitions and denominators varied, withdrawals due to adverse events were narratively synthesised and not pooled.

**Table S10. Summary of findings: incretin-based injectable strategies versus intensified insulin strategies in adults with type 2 diabetes.**

| **Outcome** | **Anticipated absolute effect** | **Relative effect / mean difference** | **Studies / participants** | **Certainty of evidence** | **Interpretation** |
| --- | --- | --- | --- | --- | --- |
| **Change in HbA1c (%)** | Not applicable for continuous outcome | **MD -0.19%** 95% CI -0.34 to -0.05 | 17 RCTs | **LOW** | Incretin-based regimens probably reduce HbA1c slightly more than intensified insulin, but the average benefit is small and heterogeneous. |
| **Hypoglycaemia, ≥1 event, trial-defined** | Comparator risk: **481 per 1,000**. Incretin-based regimen: **231 per 1,000** 95% CI 168 to 318. **Absolute effect: 250 fewer per 1,000** 95% CI 313 fewer to 163 fewer. | **RR 0.48** 95% CI 0.35 to 0.66 | 14 RCTs; n = 6,717 | **LOW** | Incretin-based regimens may substantially reduce trial-defined hypoglycaemia, but certainty is limited by open-label ascertainment and marked heterogeneity. |
| **Severe hypoglycaemia, Level 3/requiring assistance** | Comparator risk: **28 per 1,000**. Incretin-based regimen: **9 per 1,000** 95% CI 5 to 14. **Absolute effect: 19 fewer per 1,000** 95% CI 23 fewer to 14 fewer. | **RR 0.32** 95% CI 0.19 to 0.51 | 7 RCTs; n = 4,715 | **MODERATE** | Incretin-based regimens probably reduce severe hypoglycaemia compared with intensified insulin. |
| **Change in body weight (kg)** | Not applicable for continuous outcome | **MD -4.65 kg** 95% CI -5.85 to -3.44 | 16 RCTs; n = 6,687 | **LOW** | Incretin-based regimens probably reduce body weight compared with intensified insulin, but the magnitude differs substantially by regimen and drug class. |
| **Gastrointestinal adverse events** | Not pooled | Narrative synthesis: GI events, especially nausea, vomiting and diarrhoea, were generally more frequent with incretin-based regimens. | Not pooled; study-level data provided in Supplementary Tables S8. | **VERY LOW** | GI adverse events, especially nausea, vomiting, and diarrhoea, were generally more frequent with incretin-based regimens, but the exact magnitude is uncertain. |
| **Withdrawals due to adverse events** | Not pooled | Narrative synthesis: discontinuations were generally infrequent but more common in several incretin-based arms. | Not pooled; study-level data provided in Supplementary Tables S9. | **LOW** | Incretin-based regimens may increase withdrawals due to adverse events, but event numbers were low and reporting was heterogeneous. |

**Population:** Adults with type 2 diabetes requiring injectable treatment intensification or simplification.

**Intervention:** Incretin-based injectable regimens: GLP-1RA + basal insulin, fixed-ratio basal insulin/GLP-1RA combinations, dual incretin agonist regimens or related co-formulations.

**Comparator:** Intensified insulin strategies: basal-plus, basal-bolus or premixed/biphasic insulin regimens.

**Table S11: Detailed GRADE evidence profile and downgrading rationale.**

| **Outcome** | **Initial certainty** | **Risk of bias** | **Inconsistency** | **Indirectness** | **Imprecision** | **Publication bias** | **Final certainty** |
| --- | --- | --- | --- | --- | --- | --- | --- |
| **Change in HbA1c (%)** | High | **Downgrade 1**: most trials were open-label and many had RoB 2 “some concerns”; for HbA1c this is less serious than for subjective outcomes, but treatment/titration deviations could plausibly influence glycaemic outcomes. | **Downgrade 1**: substantial heterogeneity, I² = 86%, with clinically meaningful variation by regimen type and pathway. | No downgrade: population, intervention, comparator and outcome directly match the review question. | No downgrade, but interpret cautiously: CI excludes null, although the average benefit is small and may be below conventional thresholds of clinical importance. | No downgrade: funnel plot reportedly showed no clear asymmetry. | **LOW** |
| **Hypoglycaemia, ≥1 event, trial-defined** | High | **Downgrade 1**: open-label trials and variable ascertainment of symptomatic/SMBG-defined hypoglycaemia make this endpoint vulnerable to detection/reporting bias. | **Downgrade 1**: marked heterogeneity, I² = 94%, plausibly driven by different hypoglycaemia definitions, thresholds and ascertainment methods. | No downgrade. | No downgrade: RR 0.48, 95% CI 0.35 to 0.66; CI excludes no effect and supports a clear reduction. | No downgrade, but publication bias was not formally assessable because of heterogeneity. | **LOW** |
| **Severe hypoglycaemia, Level 3/requiring assistance** | High | **Downgrade 1**: severe hypoglycaemia is more objective than mild hypoglycaemia, but open-label insulin titration and treatment decisions could still influence event occurrence. | No downgrade: I² = 29%, low-to-moderate heterogeneity. | No downgrade. | No downgrade: although events were rare, the effect estimate was strong and CI excluded no effect. | No downgrade; formal assessment limited by small number of studies. | **MODERATE** |
| **Change in body weight (kg)** | High | **Downgrade 1**: body weight is objective, but open-label designs could influence lifestyle behaviour, treatment adherence, insulin titration and discontinuation patterns. | **Downgrade 1**: extreme heterogeneity, I² = 97%; direction favoured incretin-based regimens, but magnitude varied substantially by drug potency, especially dual agonist versus standard GLP-1RA/FRC regimens. | No downgrade. | No downgrade: MD -4.65 kg, 95% CI -5.85 to -3.44; CI clearly excludes no effect. | No downgrade; no clear evidence provided suggesting publication bias. | **LOW** |
| **Gastrointestinal adverse events** | High | **Downgrade 1**: nausea, vomiting, diarrhoea and GI discomfort are subjective and especially vulnerable to expectation/reporting bias in open-label trials. | **Downgrade 1**: definitions and reporting were inconsistent across trials, including symptom-specific counts versus grouped GI disorders. | No downgrade. | **Downgrade 1**: no valid pooled estimate; magnitude uncertain. | No downgrade; not formally assessable. | **VERY LOW** |
| **Withdrawals due to adverse events** | High | **Downgrade 1**: discontinuation decisions may be influenced by open-label treatment expectations and known GI tolerability profiles. | No formal downgrade if direction is broadly consistent; downgrade only if the extracted trial-level data show conflicting direction. | No downgrade. | **Downgrade 1**: event numbers are relatively low and the outcome was not pooled in the current manuscript. | No downgrade; not formally assessable. | **LOW** |
